# Comprehensive analysis of macrophage-related multigene signature in the tumor microenvironment of head and neck squamous cancer

**DOI:** 10.18632/aging.202499

**Published:** 2021-02-11

**Authors:** Bo Lin, Hao Li, Tianwen Zhang, Xin Ye, Hongyu Yang, Yuehong Shen

**Affiliations:** 1 Stomatological Center, Peking University Shenzhen Hospital, Shenzhen, Guangdong, China; 2 Guangdong Provincial High-level Clinical Key Specialty, Shenzhen, Guangdong, China; 3 Guangdong Province Engineering Research Center of Oral Disease Diagnosis and Treatment, Shenzhen, Guangdong, China; 4 Department of Pathology, Peking University Shenzhen Hospital, Shenzhen, Guangdong, China

**Keywords:** macrophage, tumor-associated macrophage, fanconi anemia complementation group E, head and neck squamous cancer, prognosis

## Abstract

Macrophages are among the most abundant cells of the tumor microenvironment in head and neck squamous cancer (HNSC). Although the marker gene sets of macrophages have been found, the mechanism by which they affect macrophages and whether they further predict the clinical outcome is unclear. In this study, a univariate COX analysis and a random forest algorithm were used to construct a prognostic model. Differential expression of the key gene, methylation status, function, and signaling pathways were further analyzed. We cross-analyzed multiple databases to detect the relationship between the most critical gene and the infiltration of multiple immune cells, as well as its impact on the prognosis of pan-cancer. FANCE is recognized as hub gene by different algorithms. It was overexpressed in HNSC, and high expression was predictive of better prognosis. It might promote apoptosis through the Wnt/β-catenin pathway. The expression of FANCE is inversely proportional to the infiltration of CD4 + T cells and their subsets, tumor-associated macrophages (TAMs), M2 macrophages, but positively co-expressed with M1 macrophages. In summary, FANCE was identified as the hub gene from the macrophage marker gene set, and it may improve the prognosis of HNSC patients by inhibiting lymphocytes and tumor-associated macrophages infiltration.

## INTRODUCTION

Head and neck squamous cell carcinoma (HNSC) is the sixth most common malignancy in the world [1], which predominantly develops from squamous cell epithelia [2]. The main risk factors of HNSC are associated with cigarette smoking, excessive alcohol use, and the presence of human papillomavirus (HPV). Although the overall survival (OS) and quality of life have been enhanced by improved standard treatment and supportive care, the HNSC prognosis remains poor, with a five-year OS rate of approximately 50% [3].

In recent years, some biomarkers have been used for the diagnosis of HNSC. For example, matrix metalloproteinases (MMPs), which promote the tumor invasion and metastasis, have been found to significantly increase in the serum of patients with head and neck cancer, and are considered promising biomarkers for the diagnosis of HNSC. In addition, DNA methylation is a major epigenetic change that often precedes the malignant proliferation of cells. The diagnosis of DNA methylation is of great significance for the early prediction of tumors. At present, the methylation status of genes such as p16, cyclin dependent kinase (CDKN), and stratifin (SFN) has been considered related to HNSC. However, the sensitivity and specificity of these biomarkers have been controversially reported in the literature. Considering that most HNSCs are diagnosed as advanced, the prognosis for HNSC patients is still very poor. Therefore, the development of new and specific prognostic markers for patients with HNSC is urgent.

Since immune-related mechanisms have a critical role in HNSC, immunotherapies represent a promising strategy for HNSC treatment [4, 5]. Immune checkpoints can respond to pathogens by regulating the balance of immune stimulus and inhibitory signals, or as regulators of mutant / overexpressing T cell immune responses [6, 7]. Research has demonstrated that the interaction between programmed cell death protein-1 (PD-1) and programmed cell death ligand-1 (PD-L1) is a critical immune checkpoint, and inhibiting PD-1 has been found to exhibit high treatment efficacy for melanoma and is now approved for the treatment of HNSC [8, 9]. However, current anti-PD-1 immunotherapy does not respond well in patients with advanced HNSC, and some patients show resistance [10, 11]. Additionally, several studies have reported that patients with a greater number of tumor-infiltrating lymphocytes display improved survival in HPV-positive and -negative oropharyngeal disease [12–15]. Therefore, there is an urgent need to elucidate the specific immune phenotypes of tumor-immune relationships and elucidate novel immunological targets for the treatment of HNSC.

Macrophages are the main stromal cells that make up the tumor microenvironment, accounting for half of the total weight of the tumor. They are called tumor-associated macrophages (TAMs). TAM interacts with tumor cells, changes the extracellular matrix, and facilitates its infiltration and metastasis. The prognosis of HNSC depends in part on the infiltration level of TAM, we therefore reasoned that there would be an altered expression of selective macrophage-related genes within tumour tissue and that this would be correlated with a change in patient survival. Marker genes for macrophages have been detected [16]. Comprehensive analysis of these gene signatures is conducive to understanding their roles in the occurrence and development of diseases. The random forest algorithm in machine learning has its unique advantages in data mining. By calculating the “feature importance” of variables to construct a prognosis model, we can obtain hub gene sets to provide a more in-depth view of the development and prognosis of HNSC.

**Figure 1:**
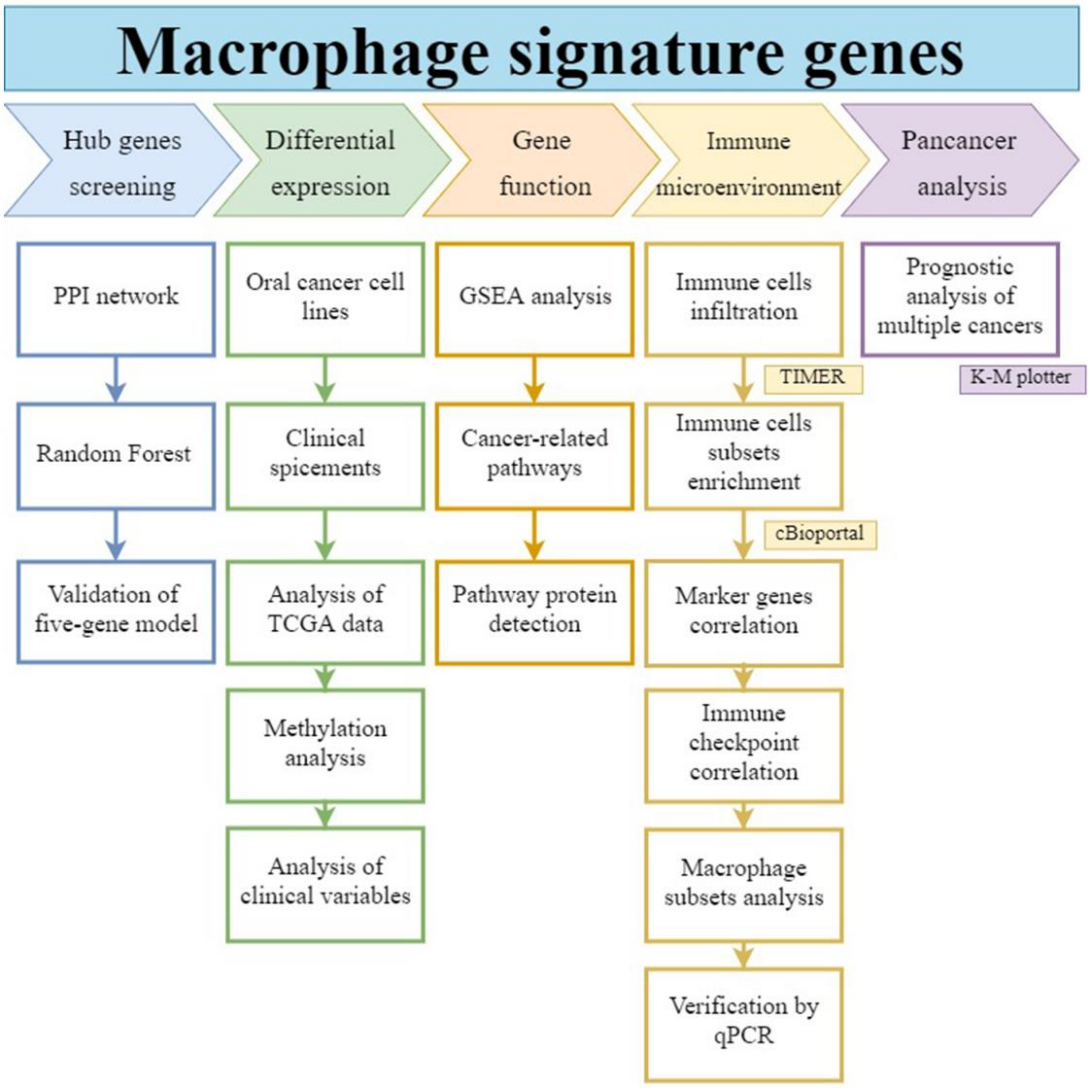
**Workflow of this study.** The hub gene was selected from macrophage-associated multigene signatures by random forest model. Its differential expression, gene function, immune infiltration relationship, and prognostic effect on pan-cancers were analyzed.

As shown in the workflow of Figure 1, in this study we operated a random forest algorithm to screen the hub genes set in the signatures of macrophages, construct a prognostic model, analyze its function and role in the immune cell network, as well as its impact on prognosis in pan-cancers.

## RESULTS

### Hub gene screening

A list of 292 macrophage marker genes was obtained from published literature. PPI network are constructed to detect gene set function and pathways. Subsequently, we constructed a random forest model to screen the hub genes.

### Construction of PPI network

**Figure 2:**
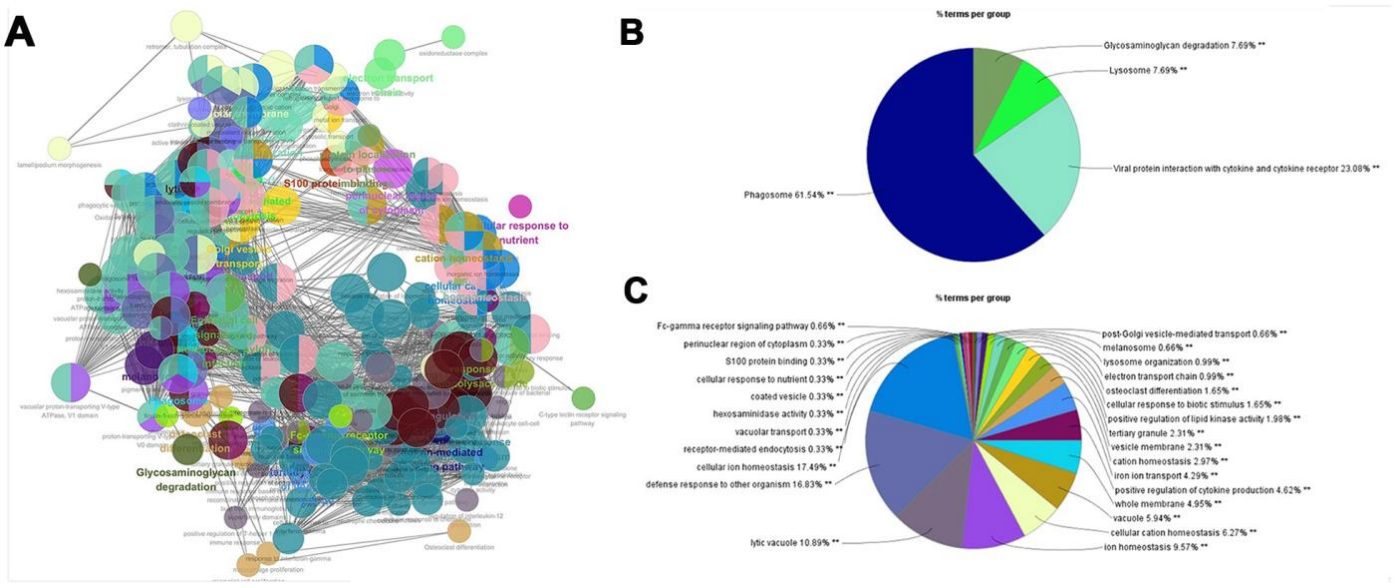
**PPI network for macrophage signature genes.** (**A**) The macrophage marker gene set was imported into STRING (https://string-db.org/) to obtain the interaction of these genes, and ClueGO app was used to map the network of enrichment results. Each node is a representative enrichment pathway, the connection line of the node indicates the number of genes shared between pathways, and the color indicates the enrichment classification of the node. (**B**) Summary of KEGG enrichment results. The function of the gene set is enriched in phagosome, viral protein interaction with cytokine and lysosome, etc. (**C**) The pie chart shows the enrichment pathway of GO, including cell components, molecular functions and biological processes. Gene function is enriched in defense response, cellular ion homeostasis, and lytic vacuole, etc.

292 genes were imported into the STRING database to construct a PPI network. In total, 292 nodes and 893 edges were presented. The ClueGo plug-in of Cytoscape was used to further analyze the functionality of the gene set, as shown in Figure 2. The functions of macrophage signature gene set were concentrated in biological processes such as phagosome, lysosome, defense response, cell ion homeostasis, and lytic vacuole. These biological processes were closely related to the phagocytic function of macrophages.

### Construction of random forest model

**Figure 3:**
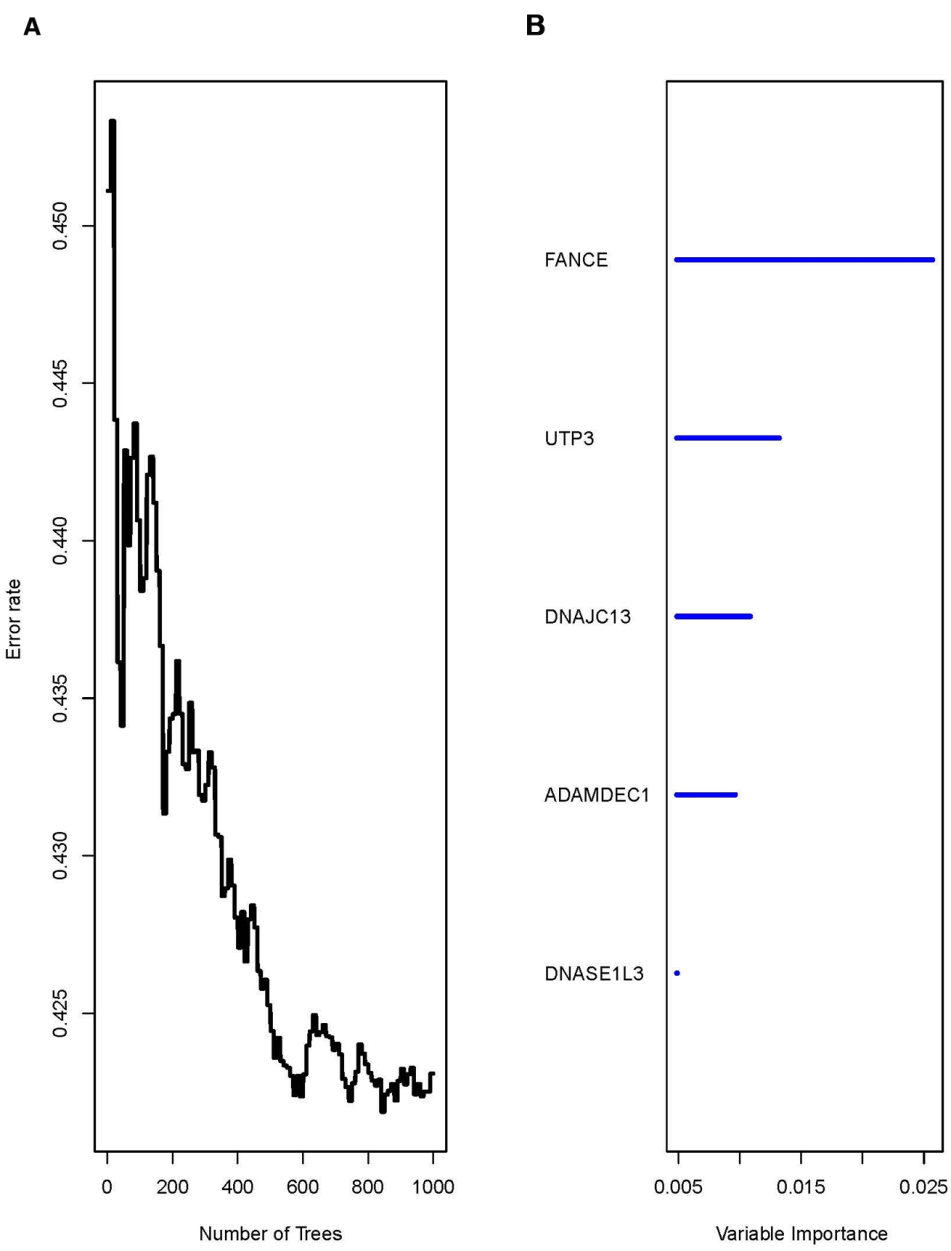
**The running result of the random forest model.** (**A**) The random forest model has the lowest error rate and tends to be stable after generating 1000 decision trees (ntree=1000). (**B**) The top five genes with highest feature important score were selected, namely FANCE, UTP3, DNAJC13, ADAMDEC1, and DNASE1L3.

Univariate COX analysis of 292 reported macrophages showed that the expression of 36 genes was significantly correlated with prognosis (*P<0.05) (Supplementary Tables 1, 2). A random forest algorithm was used to construct a prognostic model for these 36 genes. As shown in Figure 3, the model has the lowest error rate and tends to be stable when mtyr=6 and ntree=1000. The top 5 genes with highest feature important score were selected, namely fanconi anemia complementation group E (FANCE), UTP3 small subunit processome component (UTP3), DnaJ heat shock protein family (Hsp40) member C13 (DNAJC13), ADAM like decysin 1 (ADAMDEC1), and deoxyribonuclease 1 like 3 (DNASE1L3).

### Validation of prognostic models

**Figure 4:**
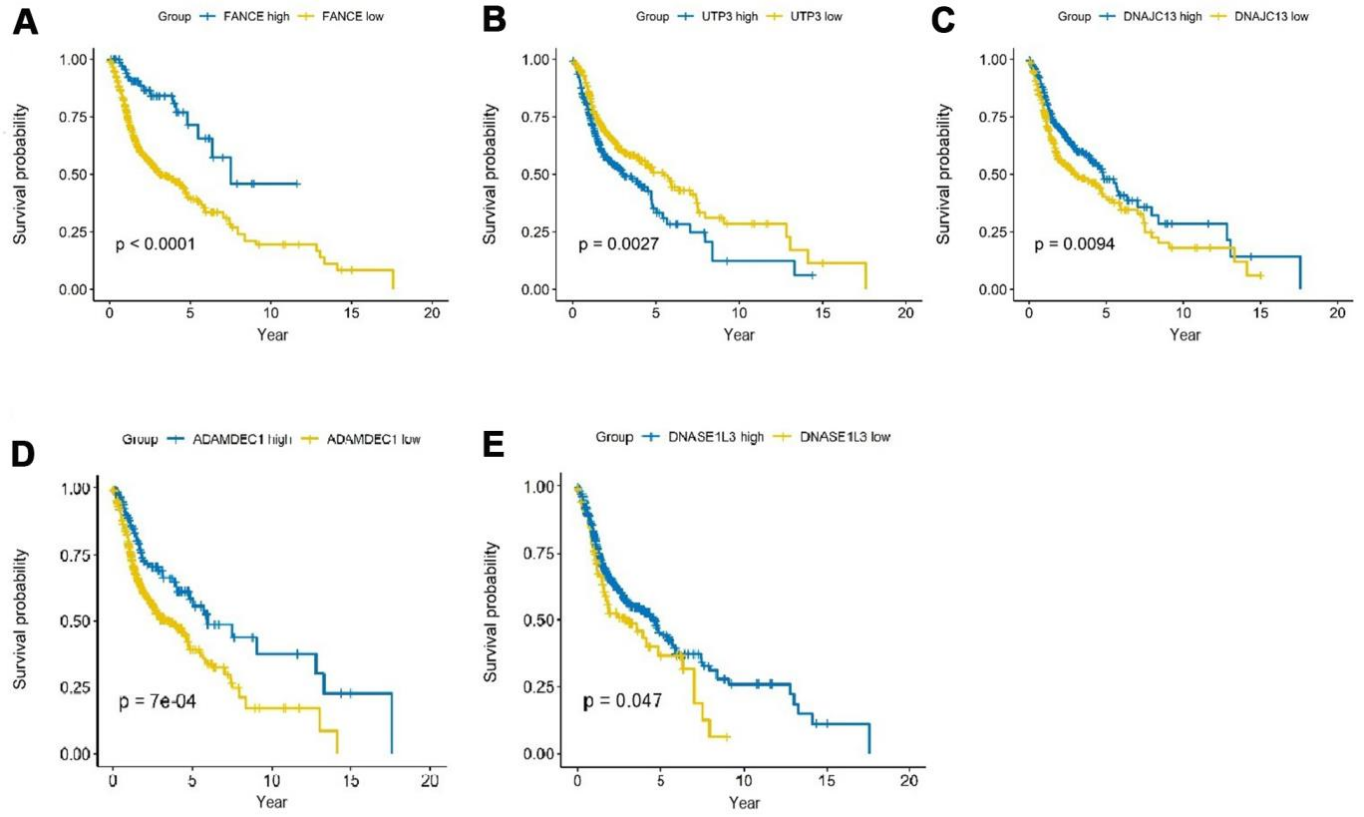
**Prognostic analysis of the top 5 genes screened by random forest in HNSC.** (**A**) HNSC patients were divided into high and low expression groups according to the optimal cutoff value. Overexpression of FANCE predicted an improved OS based on the TCGA database (*P<0.001). (**B**) Increased expression of UTP3 was associated with poorer OS (*P=2.7×10^-3^). (**C**) Overexpression of DNAJC13 predicted a better OS (*P=9.4×10^-3^). (**D**) Increased expression of ADAMDEC1 predicted an improved OS (*P=0.7×10^-3^). (**E**) Overexpression of DNASE1L3 predicted an improved OS (*P=0.047).

As shown in Figure 4, analysis of HNSC cases in TCGA showed that the expression differences of the five genes had a significant impact on the prognosis. Among them, the up-regulation of expression of FANCE, DNAJC13, ADAMDEC1 and DNASE1L3 was significantly positively associated with better 5-year survival rate, while increased expression of UTP3 was significantly correlated with a worse prognosis (all *P <0.05).

**Figure 5:**
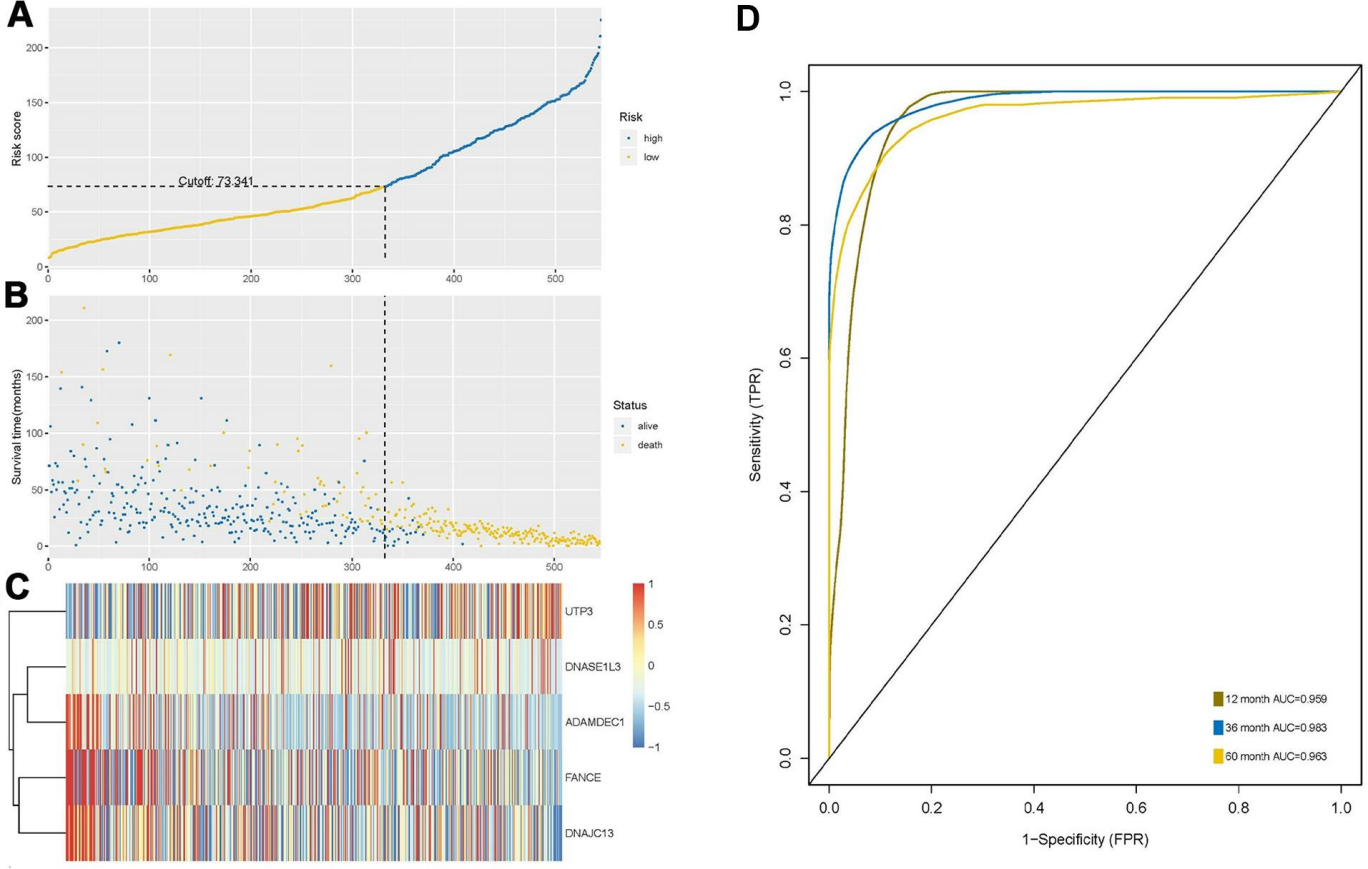
**Construction and analysis of risk score through random forest model.** (**A**) According to the optimal cutoff value of 73.341, patients were divided into high-risk and low-risk groups. (**B**) Most of the patients with long survival time are concentrated in the low-risk group (blue dots), while the high-risk group patients have significantly reduced survival time (yellow dots), indicating that this model can predict the prognosis well. (**C**) Cluster analysis of the five genes. Highly expressed UTP3 was significantly associated with high risk, while up-regulated of DNASE1L3, AMAMDEC1, FANCE, and DNAJC13 were associated with low-risk patients. (**D**) Receiver-operating characteristic (ROC) curve showed predictive ability of five-gene model, and the area under the curve (AUC) of the 1-, 3- and 5-year OS were 0.959, 0.983 and 0.963, respectively.

The risk score was calculated based on the expression levels and regression coefficients of the five target genes, and the best cutoff value was 73.341 (Figure 5A). Using this as a boundary, the high- and low-risk group can be well distinguished (Figure 5B, 5C). ROC curve showed predictive ability of the prognostic model with AUC of 1-, 3- and 5-years OS 0.959, 0.983 and 0.963, respectively (Figure 5D).

### Differential expression analysis and correlation with clinical variables

FANCE is significantly overexpressed in HNSC patients, and changes in the methylation of its specific sites may be a potential cause of upregulation. In addition, some clinicopathological variables have also been found to be closely related to the expression of FANCE.

### FANCE is up-regulated in HNSC

**Figure 6:**
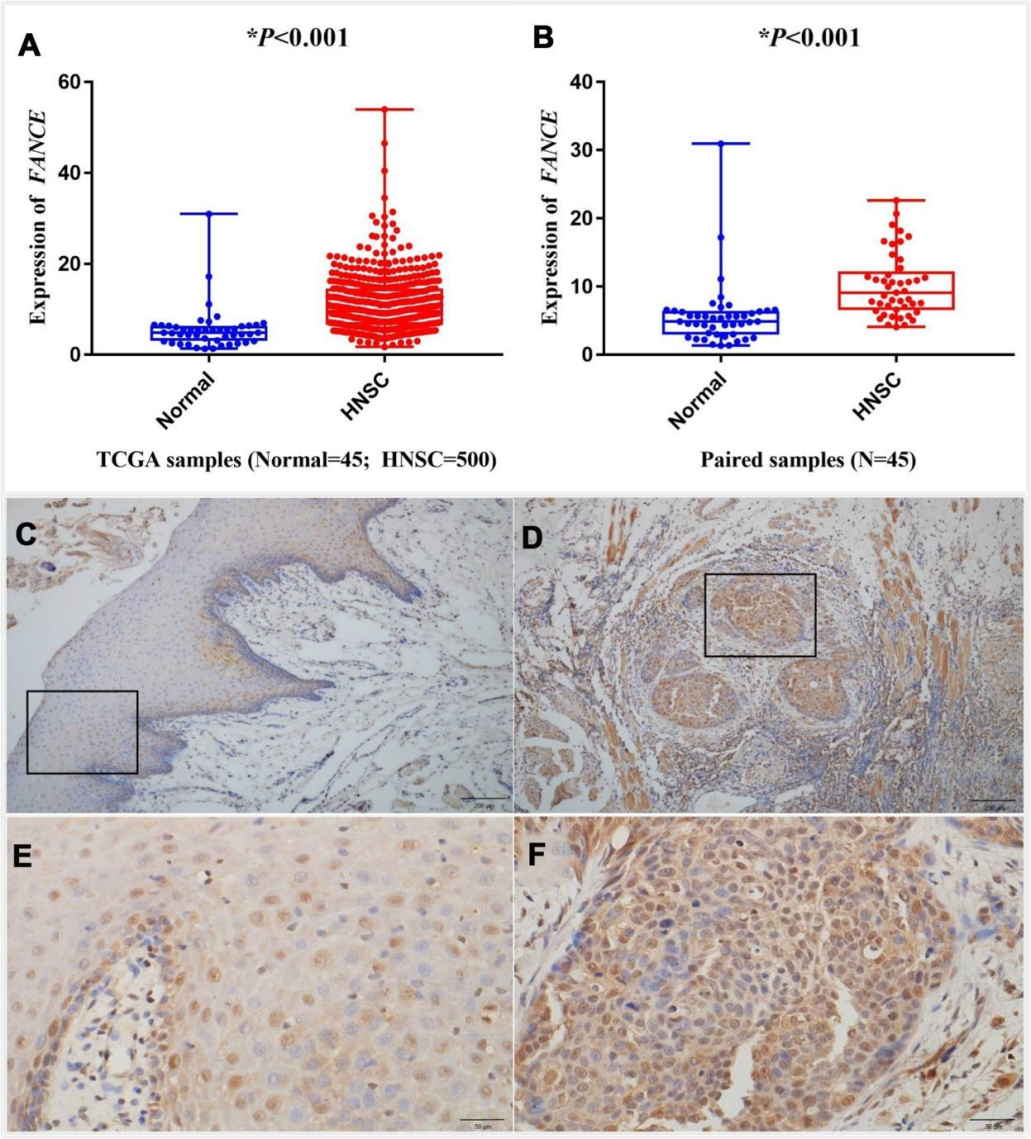
**Analysis of differential expression of FANCE in HNSC.** Representative IHC staining (200x) performed for detecting the expression of FANCE from adjacent normal tissues and tumor-tissue specimens of HNSC patients from human protein atlas (https://www.proteinatlas.org/). (**A**) FANCE expression was compared between HNSC tissues and normal tissues based on the TCGA database. FANCE expression in HNSC (n = 500) was significantly increased than that in normal patients (n = 45). (**B**) The level of FANCE expression was compared between the paired samples. Matching cancer tissues and adjacent normal tissues of the same patient also showed that FANCE expression was significantly up-regulated in cancer tissues. (**C**–**F**) C and E showed normal tissue, while D and F were HNSC tissue. Immunohistochemical staining was performed to detecting the expression of FANCE in adjacent normal tissues (C, x200 and E, x400) and tumor tissue specimens (D, x200 and F, x400) of patients with HNSC.

The differential expression between the tumor and normal tissues for FANCE in HNSC of TCGA is shown in Figure 6A, 6B. The results indicated that FANCE was overexpressed in HNSC samples (*P<0.001) and paired samples (*P<0.001). Expression data of FANCE and paired data can be obtained in the Supplementary files (Supplementary Tables 3, 4).

Oral squamous cell carcinoma (OSCC) specimens from 15 pathologically confirmed HNSC patients aged 45 to 87 were included in the study. Immunohistochemical staining of HNSC tissue specimens and adjacent normal tissues revealed that FANCE was strongly stained in tumor tissues (Figure 6D, 6F), but the staining intensity was lower in adjacent normal tissues (Figure 6C, 6E).

### Methylation analysis

**Figure 7:**
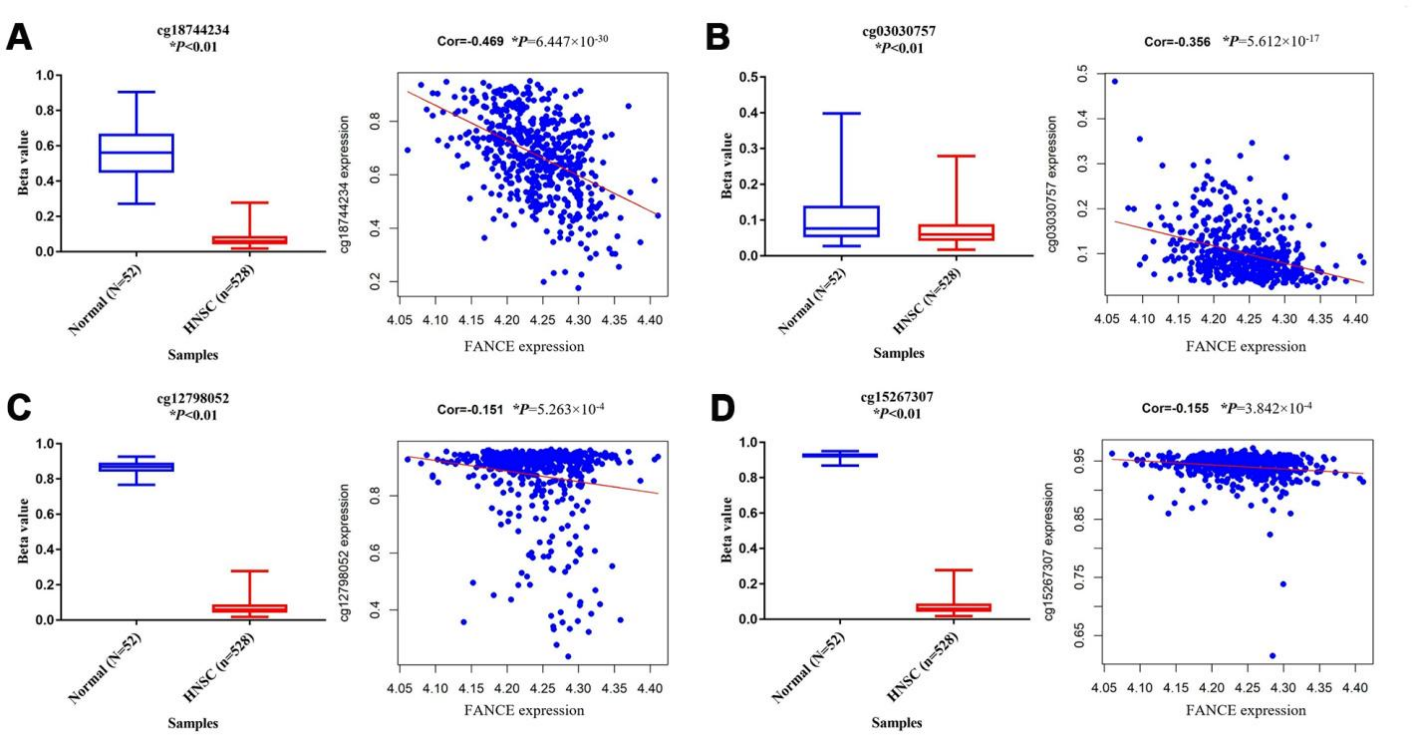
**Analysis of methylation status and expression in FANCE.** (**A**) The methylation site cg18744234 is hypomethylated in tumor samples (n = 528) and is negatively correlated with the expression of FANCE (*P<0.001). (**B**) The methylation site cg03030757 is hypomethylated in tumor samples and is negatively correlated with the expression of FANCE (*P<0.001). (**C**) Cg12798052 was significantly hypomethylated in tumor samples and was negatively correlated with the expression of FANCE (*P<0.001). (**D**) Cg15267307 was significantly hypomethylated in tumor samples and was negatively correlated with the expression of FANCE (*P<0.001).

In order to explore the possible reasons for the up-regulation of FANCE in HNSC, the relationship between its methylation status and its expression was further analyzed. A total of 10 methylation sites were found in the FANCE gene. Among them, cg12798052 in the promoter region, cg03030757 and cg18744234 in the gene enhancement region, and cg15267307 in the transcription region were detected with significant hypermethylation (Figure 7). Additionally, they are also significantly negatively correlated with the expression of FANCE (all *P<0.05). We also observed that the hypermethylation status of cg09490277 was significantly positively correlated with the expression of FANCE, while no significant effect of methylation status at other five methylation sites on the expression of FANCE was observed. Relationship between methylation sites and expression of FANCE could be available from Supplementary Table 5.

### Association with FANCE expression and clinicopathologic variables

As shown in Table 1, the increased expression of FANCE was significantly correlated with tumor origin (oropharynx vs. oral cavity; n=577, *P=5.867×10^-6^), pathologic T classification (T I&II vs. III&IV; n=522, *P=3.44×10^-4^), HPV infection status (positive vs. negative; n=115, *P=1.78×10^-4^), perineural invasion (negative vs. positive; n=410, *P=0.014) and smoking (positive vs. negative; n=577, *P=0.004). However, no significant differences between FANCE expression and age, lymph node invasion, clinical stage, lymphovascular invasion and histologic grade were found.

**Table 1:** Correlation between *FANCE* expression and the clinicopathological characteristics of HNSC patients (logistic regression).

**Clinical Characteristic**	**Total (N)**	**Odds ratio in *FANCE* expression**	***P***
Age (continuous)	546	0.987 (0.974-1.001)	0.072
Tumor origin (Oropharynx vs. Oral cavity)	546	2.300 (1.609-3.309)	5.867×10^-6^
Lymph nodes (Positive vs. Negative)	469	1.010 (0.700-1.459)	0.956
Pathologic T classification (T1&T2 vs. T3&T4)	546	1.916 (1.344-2.742)	3.44×10^-4^
Stage (I&II vs. III&IV)	546	1.630 (0.613-4.856)	0.347
HPV (Positive vs. Negative)	115	6.111 (2.479-16.818)	1.78×10^-4^
lymphovascular invasion (Positive vs. Negative)	395	1.101 (0.729-1.664)	0.647
Perineural invasion (Positive vs. Negative)	410	0.611 (0.413-0.902)	0.014
Histologic grade (G3&G4 vs. G1&G2)	546	1.038 (0.710-1.519)	0.846
Smoke (Positive vs. Negative)	546	1.623 (1.168-2.261)	0.004

### Functional analysis of FANCE

The overexpression of FANCE is related to improved prognosis of HNSC patients, so we further explored the function and possible signal pathways of FANCE through gene set enrichment analysis.

### GSEA identifies a FANCE-related signaling pathway

**Figure 8:**
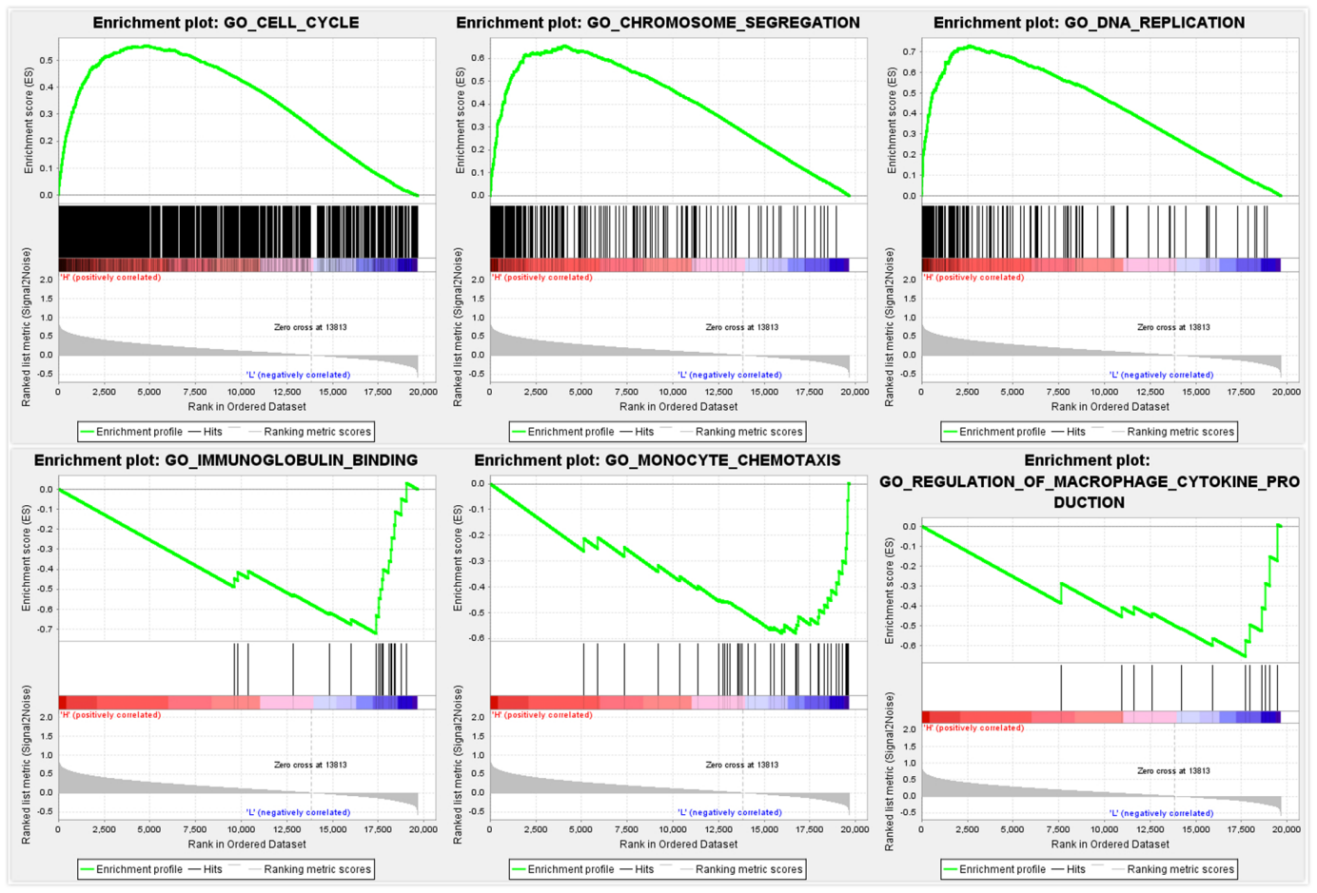
**GSEA identifies a FANCE-related signaling pathway.** Cell cycle, DNA repair, and apoptosis signaling pathways are differentially enriched in the high FANCE expression phenotype. Immunoglobulin binding, monocyte chemotaxis and regulation of macrophage cytokine production are enriched in the FANCE down-regulation phenotype.

GO and KEGG pathway enrichment analysis results show that 1052 and 28 gene sets were significantly enriched when the nominal p-value was <5%. As shown in Figure 8, multiple anti-tumor-associated biological processes such as cell cycle, DNA repair and apoptotic signaling pathways, and so on, were enriched in response to increased FANCE. In addition, with regards to GO pathways, the down-regulated FANCE were associated with immunoglobulin binding, monocyte chemotaxis and regulation of macrophage cytokine production are enriched in the FANCE down-regulation phenotype.

### Relationship between FANCE and cancer-related pathways

The enriched signal pathway suggested in the GSEA results was selected for further ssGSEA analysis. The correlation between FANCE and the signal pathway was calculated from the known gene set. The results are shown in Table 2. Fanconi anemia, cell cycle, mismatch repair, DNA damage repair, DNA replication, homologous recombination, nucleotide excision repair, WNT target and antigen processing machinery, were the primary enriched pathways. EMT2, EMT3, pan-fibroblast TGF-β response signature (panFTBRS), and angiogenesis were found to be significantly negatively correlated with the expression of FANCE.

**Table 2:** Relationship between *FANCE* expression and classic cancer-related pathways / biological processes.

**Cancer-related pathways / biological processes**	**Correlation coefficient**	***P***
Fanconi anemia	0.612	<0.001
Cell cycle	0.595	<0.001
Mismatch repair	0.557	<0.001
DNA damage repair	0.541	<0.001
DNA replication	0.54	<0.001
Homologous recombination	0.489	<0.001
Nucleotide excision repair	0.418	<0.001
WNT target	0.164	<0.001
Antigen processing machinery	0.092	0.031
EMT1^*^	-0.019	0.66
CD8 T effector	-0.03	0.482
Immune checkpoint	-0.066	0.122
EMT2	-0.124	0.004
Pan.F.TBRS^#^	-0.218	<0.001
EMT3	-0.287	<0.001
Angiogenesis	-0.299	<0.001

^*^:EMT: epithelial to mesenchymal transition; ^#^:Pan.F.TBRS: *pan*-fibroblast TGFβ response signature.

### FANCE involved in OSCC cell WNT pathways

**Figure 9:**
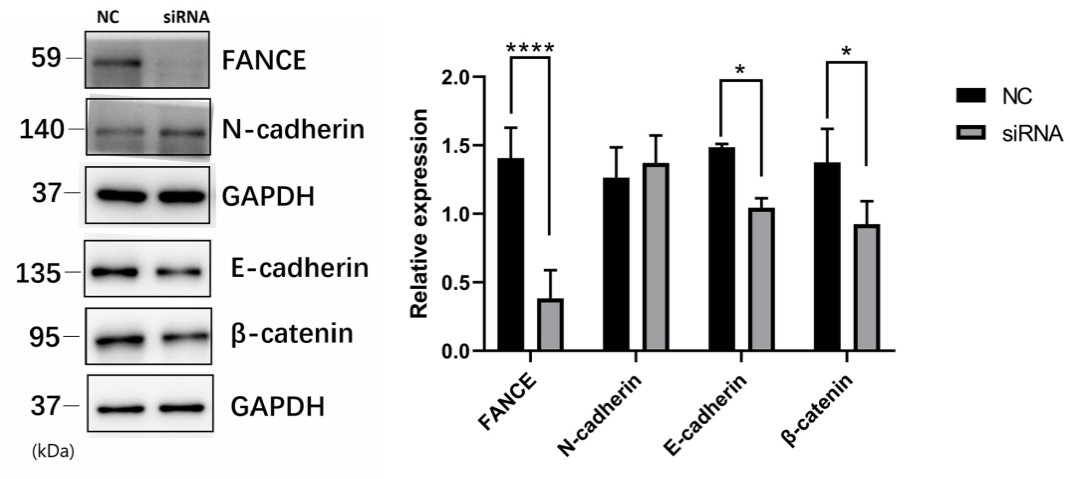
**The relationship between FANCE and key proteins in the Wnt / β-catenin pathway by Western blotting.** Compared with control OSCC cells, FANCE depleted cells showed decreased expression of E-cadherin and β-catenin.

To investigate the molecular basis of FANCE regulation in OSCC cells, we examined the expression of several key proteins. Precious studies have shown that Wnt / β-catenin pathway is crucial for cell proliferation and apoptosis. Here, to evaluate the Wnt process, we suppressed FANCE expression and detected Wnt markers by performing Western blotting. As compared to control OSCC cells, FANCE depleted cells showed decreased expression of E-cadherin and β-catenin (Figure 9), which indicates that FANCE functions in the Wnt / β-catenin pathway process in OSCC cells. The results indicate that FANCE participates in the Wnt / β-catenin process and affects the expression levels of proteins that are crucial for cell cycle progression and apoptosis.

### The role of FANCE in immune microenvironment

The correlation between the expression of FANCE and a variety of immune cells and immune checkpoint marker genes was analyzed.

### Relationship between FANCE and infiltration of immune cells

**Figure 10:**
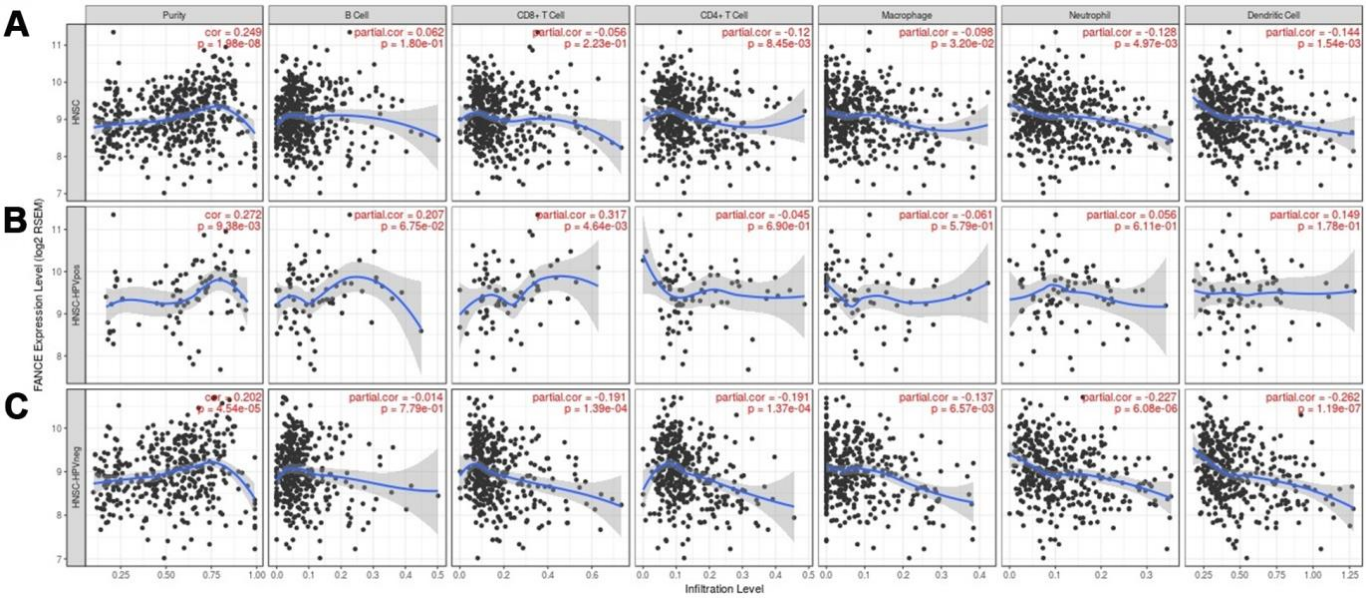
**Correlation between the level of FANCE expression and immune infiltration in HNSC obtained from the TIMER database (https://cistrome.shinyapps.io/timer/).** (**A**) FANCE expression was significantly negatively correlated with infiltration of CD4+ T cells, macrophages, neutrophils, and dendritic cells in HNSC. Tumor purity and FANCE were found to have a significant positive correlation, while B cell and CD8 + T cell infiltration levels and FANCE expression were not significantly correlated. (**B**, **C**) Similar negative correlations were observed with the level of infiltrating lymphocytes in HPV-negative HNSC samples, but no significant correlation was found in HPV-positive patients.

We found that the level of FANCE expression correlated with high levels of immune infiltration in four types of immune cells in the TIMER dataset as well as tumor purity. Figure 10 shows that FANCE expression was significantly negatively correlated with CD4+ T cell, neutrophil, macrophage, and DC infiltration. Similarly, in HPV-negative HNSC samples, the infiltration levels of these four lymphocytes and CD8 + T cells were also found to be significantly correlated with the expression of FANCE. However, positive correlation was found between CD4 + T cell infiltration and FANCE expression in HPV-negative patients.

### Relationship between FANCE and subgroups of immune cells

**Figure 11:**
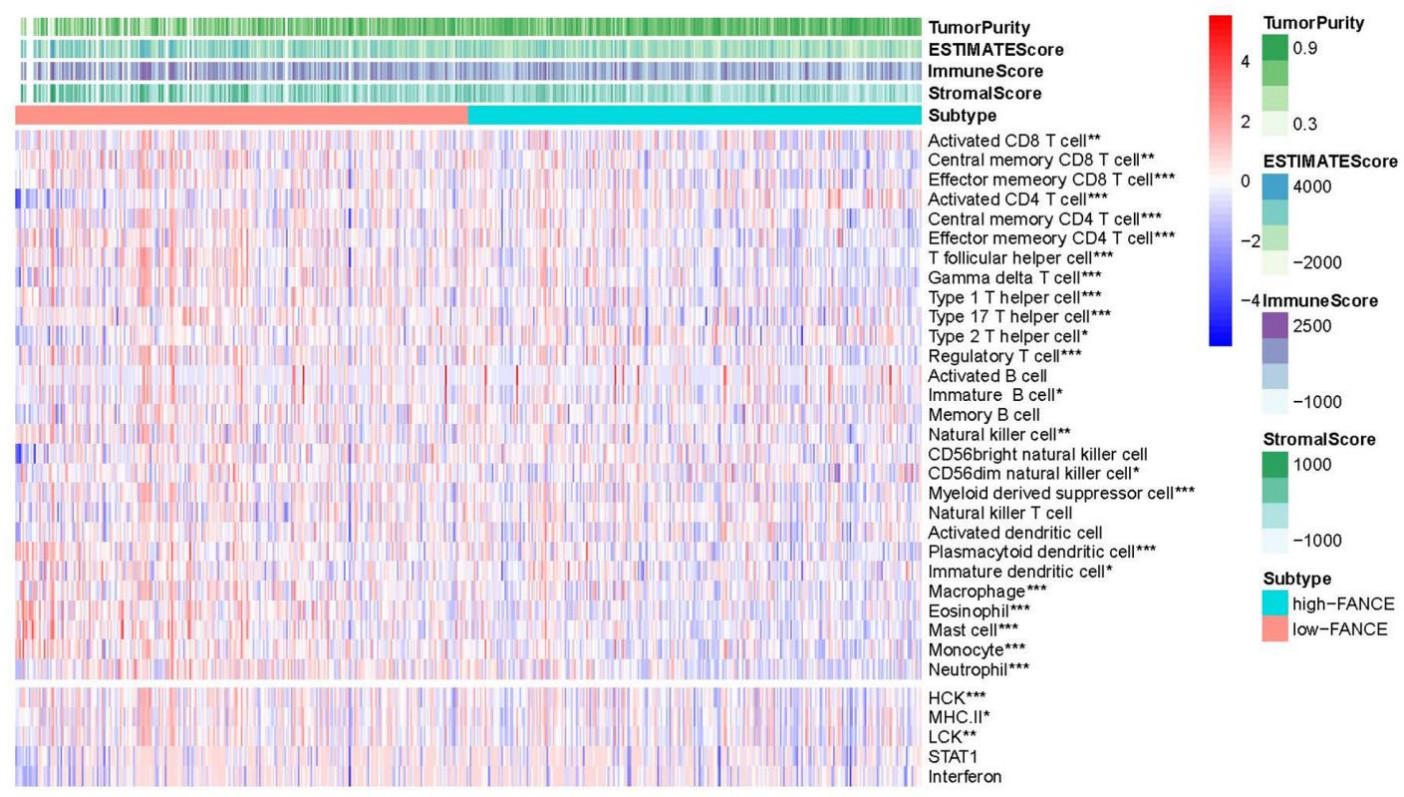
**Correlation between the level of FANCE expression with lymphocyte abundance in HNSC.** FANCE expression was significantly associated with an abundance of Act CD8, Tcm CD8, Tem CD8, Act CD4, Tcm CD4, Tem CD4, Tfh, Tgd, Th1, Th17, Th2, Treg, Imm B, NK, CD56dim, MDSCs, pDCs, iDCs, MHC II, LCK, macrophages, eosinophils, Mast, monocytes, and neutrophils (all *P< 0.05).

Figure 11 shows that FANCE expression was significantly negatively associated with an abundance of Act CD8, Tcm CD8, Tem CD8, Act CD4, Tcm CD4, Tem CD4, Tfh, Tgd, Th1, Th17, Th2, Treg, Imm B, NK, CD56dim, MDSCs, pDCs, iDCs, MHC II, LCK, macrophages, eosinophils, Mast, monocytes, and neutrophils (all *P* values < 0.05). The scoring data of 33 kinds of immune cells are based on the results of ESTIMATE calculation could be obtained from Supplementary Table 6.

### FANCE expression and immune checkpoint correlation analysis

**Figure 12:**
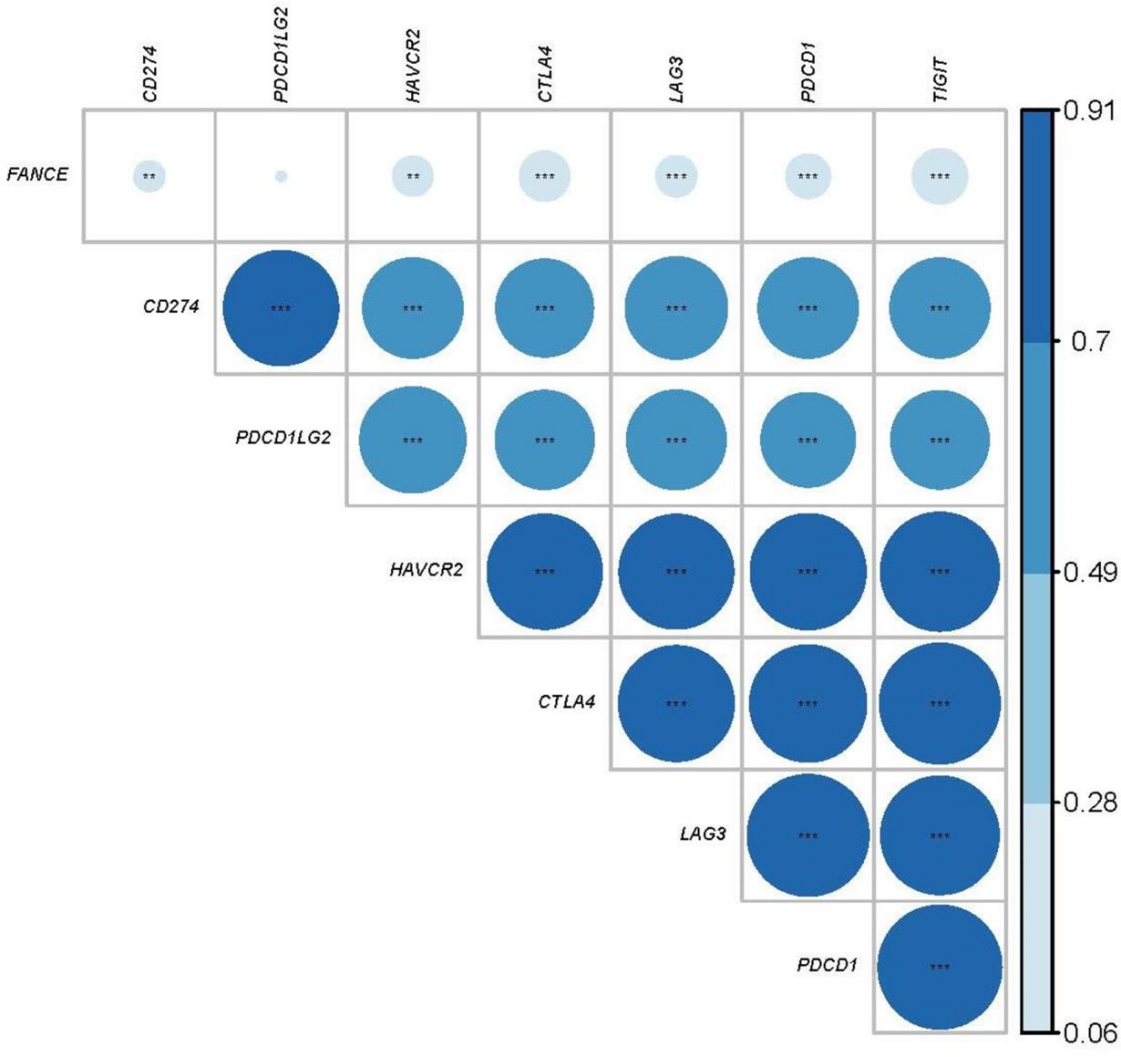
**Relationship between FANCE and immune checkpoint related genes.** The larger the area of the circle, the darker the color and the greater the correlation. The asterisk in the circle indicates statistical significance. The results showed that there was a significant correlation (*P<0.05) between the expression of FANCE and reported immune-related genes (CD274, HAVCR2, CTLA4, LAG3, PDCD1, TIGIT), but no significant co-expression between PDCD1LG2 and FANCE relationship.

We further calculated the co-expression between FANCE and the specific genes of the immune checkpoints that have been reported in the literature. Programmed Cell Death 1 Ligand 1 (*CD274*), Hepatitis A Virus Cellular Receptor 2 (*HAVCR2*), Cytotoxic T-Lymphocyte Associated Protein 4 (*CTLA4*), Lymphocyte Activating 3 (*LAG3*), Programmed Cell Death 1 (*PDCD1*) and T Cell Immunoreceptor With Ig And ITIM Domains (*TIGIT*) are recognized as immunological checkpoint-specific genes [17–23]. Figure 12 shows that FANCE is significantly associated with the expression of these genes (*P < 0.05). However, no significant correlation was found between Programmed Cell Death 1 Ligand 2 (*PDCD1LG2*) and FANCE (*P*=0.136). Expression data for FANCE and immune checkpoint marker genes were provided in Supplementary Table 7.

### Correlation between FANCE and immune cells infiltration on pan-cancers

As a marker gene for macrophages, FANCE was found to correlate significantly with macrophage infiltration levels in 15 types of cancers from TCGA data. As shown in Table 3, in lung squamous cell carcinoma (LUSC), lung adenocarcinoma (LUAD), esophageal carcinoma (ESCA), sarcoma (SARC), stomach adenocarcinoma (STAD), glioblastoma multiforme (GBM), brain lower grade glioma (LGG), uterine corpus endometrial carcinoma (UCEC), rectum adenocarcinoma (READ), cervical squamous cell carcinoma and endocervical adenocarcinoma (CESC), colon adenocarcinoma (COAD), testicular germ cell tumors and uveal melanoma (TGCT), FANCE was found to be significantly inversely related to macrophage infiltration levels. In 7 of these cancers, the expression of FANCE was significantly negatively correlated with the infiltration level of CD4 + T cells. In addition, in six and five of these cancer data, FANCE was significantly negatively correlated with neutrophil and dendritic cell infiltration, respectively. Overall, in LUSC and STAD, FANCE showed a correlation with immune cell infiltration consistent with HNSC. Interestingly, the relationship between FANCE and immune cells in liver hepatocellular carcinoma (LHC), prostate adenocarcinoma (PRAD), breast invasive carcinoma (BRCA), and kidney renal papillary cell carcinoma (KIRP) appears to be completely opposite to that in HNSC.

**Table 3:** Relationship between expression of FANCE and immune cell infiltration in various cancers.

**Ca types**	**purity**	**B cell**	**CD8+**	**CD4+**	**macrophage**	**neutrophil**	**Dendritic**
ACC	-0.016	**0.314**	0.131	0.084	0.154	**0.337**	**0.477**
BLCA	0.042	-0.006	**0.263**	-0.052	0.072	**0.212**	**0.249**
BRCA	0.032	**0.108**	0.01	**0.144**	**-0.167**	**0.096**	**0.123**
CESC	0.027	-0.033	0.035	0.098	**-0.242**	**0.136**	**0.177**
CHOL	0.04	**0.345**	0.176	0.038	-0.15	**-0.205**	**0.247**
COAD	-0.003	-0.01	-0.047	-0.055	**-0.127**	**0.015**	**0.017**
DLBC	0.169	0.118	-0.164	0.16	0.262	-0.306	-0.022
ESCA	0.107	**-0.195**	**-0.259**	**-0.183**	**-0.198**	**-0.176**	0.101
GBM	**0.425**	**-0.106**	-0.053	**-0.12**	**-0.114**	0.001	0.051
HNSC	**0.249**	0.062	-0.056	**-0.12**	**-0.098**	**-0.128**	**-0.144**
KCH	0.237	**-0.363**	**-0.286**	0.14	-0.148	-0.168	**-0.273**
KIRC	**-0.111**	**-0.116**	**0.223**	**0.213**	0.066	**0.119**	0.072
KIRP	-0.026	**0.168**	0.053	**0.215**	**-0.197**	**0.304**	**0.185**
LGG	**0.488**	**-0.142**	**-0.196**	-0.031	**-0.109**	**-0.2**	**-0.097**
LHC	**0.175**	**0.289**	**0.275**	**0.242**	**0.349**	**0.276**	**0.378**
LUAD	**0.122**	-0.085	-0.023	**-0.097**	**-0.099**	-0.063	-0.065
LUSC	**0.283**	**-0.215**	**-0.302**	**-0.202**	**-0.176**	**-0.315**	**-0.357**
MESD	**0.251**	**0.23**	-0.004	**0.245**	0.061	-0.139	0.107
OV	**0.21**	-0.022	-0.062	**0.078**	-0.051	-0.01	-0.027
PAAD	**0.265**	0.019	-0.087	-0.042	**-0.162**	-0.08	-0.005
PCPG	**0.155**	-0.131	0.01	0.068	-0.075	-0.059	**-0.153**
PRAD	**-0.104**	**0.118**	0.068	**0.228**	**0.134**	**0.132**	**0.166**
READ	-0.027	-0.088	-0.157	-0.072	**-0.259**	-0.112	-0.136
SARC	**0.421**	0.099	0.033	**-0.211**	**-0.215**	-0.115	-0.112
SKCM	**0.176**	0.003	**-0.113**	0.001	-0.013	**-0.143**	-0.021
STAD	**0.136**	-0.073	**-0.175**	**-0.146**	**-0.406**	**-0.207**	**-0.291**
TGCT	**-0.328**	**0.293**	**0.292**	-0.038	-0.139	-0.044	**0.271**
THCA	**-0.059**	**0.275**	-0.023	0.059	**0.115**	**0.09**	**0.186**
THYM	0.138	**-0.422**	**-0.3**	**-0.484**	-0.067	0.132	**-0.471**
UCEC	**0.152**	**-0.126**	**-0.134**	-0.024	**-0.224**	**0.119**	-0.107
USC	**0.368**	-0.131	-0.076	-0.047	-0.02	**-0.36**	-0.131
UVM	**-0.211**	-0.002	-0.203	-0.04	**-0.252**	**0.25**	-0.03

Bold font: *P*<0.05. ACC: Adrenocortical carcinoma; BLCA: Bladder Urothelial Carcinoma; BRCA: Breast invasive carcinoma; CESC: Cervical squamous cell carcinoma and endocervical adenocarcinoma; CHOL: Cholangio carcinoma; COAD: Colon adenocarcinoma; DLBC: Lymphoid Neoplasm Diffuse Large B-cell Lymphoma; ESCA: Esophageal carcinoma; GBM: Glioblastoma multiforme; HNSC: Head and Neck squamous cell carcinoma; KICH: Kidney Chromophobe; KIRC: Kidney renal clear cell carcinoma; KIRP: Kidney renal papillary cell carcinoma; LAML: Acute Myeloid Leukemia; LGG: Brain Lower Grade Glioma; LIHC: Liver hepatocellular carcinoma; LUAD: Lung adenocarcinoma; LUSC: Lung squamous cell carcinoma; MESO: Mesothelioma; OV: Ovarian serous cystadenocarcinoma; PAAD: Pancreatic adenocarcinoma; PCPG: Pheochromocytoma and Paraganglioma; PRAD: Prostate adenocarcinoma; READ: Rectum adenocarcinoma; SARC: Sarcoma; SKCM: Skin Cutaneous Melanoma; STAD: Stomach adenocarcinoma; TGCT: Testicular Germ Cell Tumors; THCA: Thyroid carcinoma; THYM: Thymoma; UCEC: Uterine Corpus Endometrial Carcinoma; UCS: Uterine Carcinosarcoma; UVM: Uveal Melanoma.

### FANCE expression and immune marker correlation analysis

**Figure 13:**
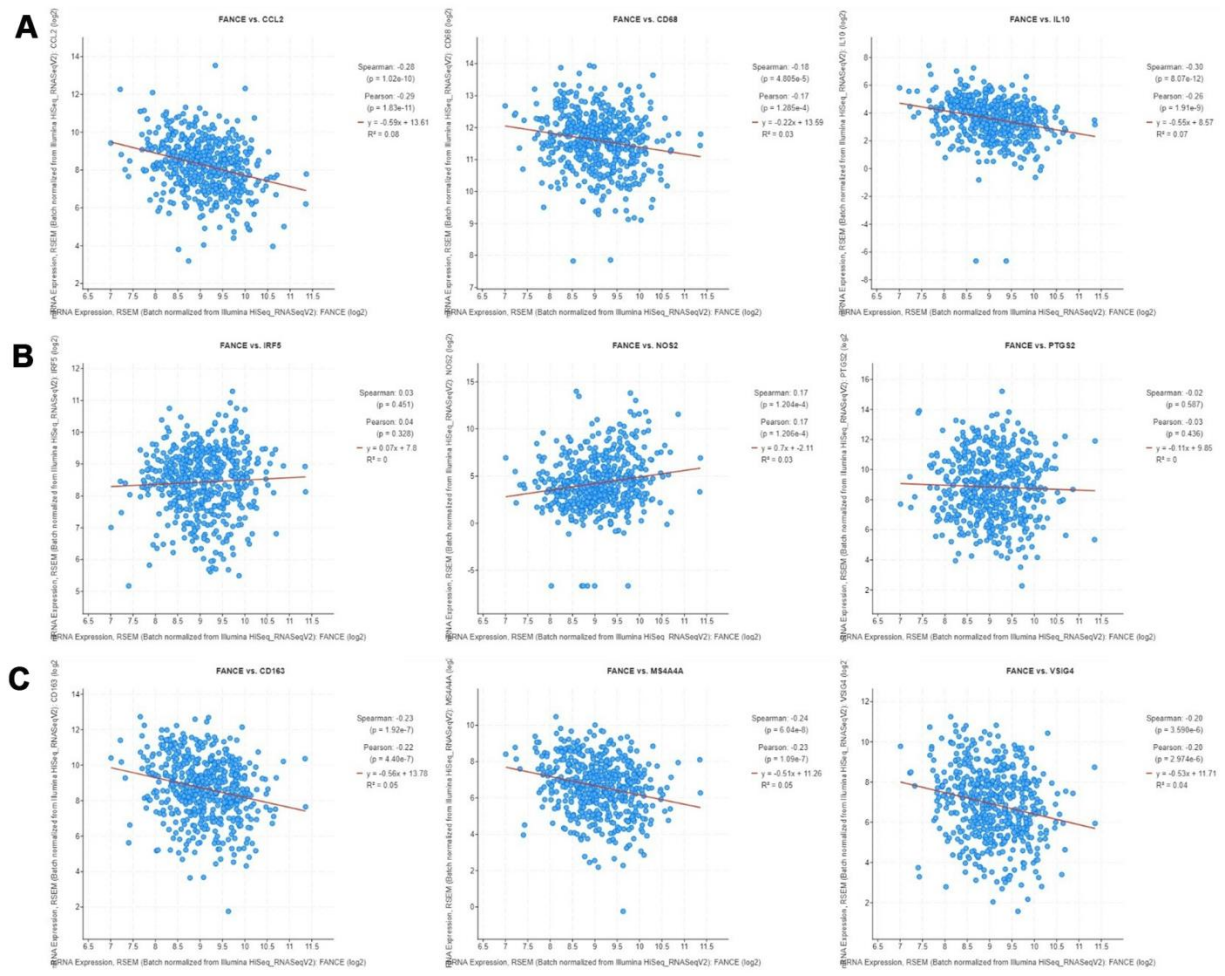
**Correlation analysis between FANCE and macrophage marker genes in cBioportal (https://www.cbioportal.org/).** (**A**) TAM marker genes, including CCL2, CD68 and IL10, have a significant negative correlation with FANCE (all *P<0.05). (**B**) IRF5, NOS2 and PTGS2 are marker genes of M1 macrophages. There was a significantly positive co-expression of FANCE and NOS2 (*P= 1.204×10^-4^), but no significant co-expression relationship was found in IRF5 and PTGS2. (**C**) Marker genes of M2 macrophages, including CD163, MS4A4A and VSIG4, had a significant negative correlation with FANCE (all *P<0.05).

The correlation between the expression of FANCE on M1, M2 macrophage phenotypes and TAM was calculated in HNSC. As shown in Figure 13, TAM marker genes, especially hyaluronan mediated motility receptor (CD68), Interleukin 10 (IL10) and chemokine (C-C motif) ligand 2 (CCL2), as well as CD163, V-set and immunoglobulin domain containing 4 (*VSIG4*) and membrane spanning 4-domains A4A (*MS4A4A*) of M2 macrophage phenotype, were significantly negatively correlated with the expression of FANCE in HNSC (*P<0.05). The expression of M1 marker genes, such as nitric oxide synthase 2 (NOS2), interferon regulatory factor 5 (*IRF5*), prostaglandin-endoperoxide synthase 2 (*PTGS2*), showed generally positively correlated with the expression of FANCE.

**Figure 14:**
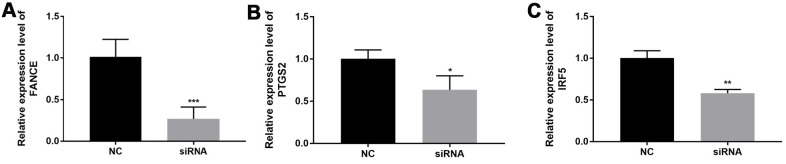
**The relationship between FANCE and M1 macrophage marker genes.** (**A**) OSCC line CAL-27 transfected with FANCE siRNA showed a significant down-regulation of FANCE expression. (**B**) The down-regulation of FANCE is associated with a decrease in the expression of prostaglandin-endoperoxide synthase 2 (PTGS2). (**C**) The down-regulation of FANCE was significantly related to the low expression of interferon regulatory factor 5 (IRF5).

In order to verify relationship between FANCE expression and macrophages, OSCC line CAL-27 were transfected with FANCE siRNA. Compared to control OSCC cells, the marker gene of M1 macrophages (PTGS2 and IRF5) in FANCE depleted cells was significantly down-regulated (Figure 14).

In addition, the expression of FANCE also showed a significant negative correlation with the marker genes of CD4 + T cell subsets such as Treg, Th1, Th2, and Th17. Similar to the TIMER results, we also observed a significant negative correlation between the expression of FANCE and the marker genes of neutrophils and dendritic cells (Table 4).

**Table 4:** Correlation analysis between *FANCE* and relate genes and markers of immune cells in cBioportal.

	**Correlated Gene**	**Spearman's correlation**	***P***
CD8+ T cell	*CD8A*	-0.109	*0.013
	*CD8B*	-0.079	0.073
T cell (general)	*CD3D*	-0.136	1.836×10^-3^
	*CD3E*	-0.146	*8.144×10^-4^
	*CD2*	-0.150	*5.687×10^-4^
B cell	*CD19*	-0.0174	0.692
	*CD79A*	-0.021	0.626
Monocyte	*CD86*	-0.247	*1.08×10^-8^
	*CSF1R*	-0.267	*5.68×10^-10^
TAM	*CCL2*	-0.282	*5.03×10^-11^
	*CD68*	-0.184	*2.380×10^-5^
	*IL10*	-0.301	*2.13×10^-12^
M1 Macrophage	*NOS2*	0.163	*1.901×10^-4^
	*IRF5*	0.045	0.301
	*PTGS2*	-0.025	0.562
M2 Macrophage	*CD163*	-0.280	*7.07×10^-11^
	*VSIG4*	-0.207	*1.940×10^-6^
	*MS4A4A*	-0.239	*3.08×10^-8^
Neutrophils	*CEACAM8*	0.149	*6.366×10^-4^
	*ITGAM*	-0.084	0.055
	*CCR7*	-0.197	*5.684×10^-6^
Natural killer cell	*KIR2DL1*	0.020	0.652
	*KIR2DL3*	-0.081	0.064
	*KIR2DL4*	0.039	0.380
	*KIR3DL1*	0.019	0.674
	*KIR3DL2*	-0.064	0.146
	*KIR3DL3*	0.051	0.242
	*KIR2DS4*	0.022	0.615
Dendritic cell	*HLA-DPB1*	-0.157	*3.302×10^-4^
	*HLA-DQB1*	-0.072	0.099
	*HLA-DRA*	-0.133	*2.308×10^-3^
	*HLA-DPA1*	-0.142	*1.146×10^-3^
	*CD1C*	-0.149	*6.508×10^-4^
	*ITGAX*	-0.176	*5.109×10^-5^
	*NRP1*	-0.297	*4.48×10^-12^
Th1	*TBX21*	-0.0989	*0.024
	*STAT4*	-0.176	*5.460×10^-5^
	*STAT1*	-0.172	*7.909×10^-5^
	*IFNG*	-0.066	0.132
	*TNF*	-0.100	*0.022
Th2	*GATA3*	-0.229	*1.200×10^-7^
	*STAT6*	0.0458	0.296
	*STAT5A*	0.098	*0.026
	*IL13*	-0.110	*0.012
Tfh	*BCL6*	0.265	*7.24×10^-10^
	*IL21*	-0.121	*5.637×10^-3^
Th17	*STAT3*	-0.033	0.446
	*IL17A*	-0.064	0.143
Treg	*FOXP3*	-0.151	*5.486×10^-4^
	*CCR8*	-0.145	*8.847×10^-4^
	*STAT5B*	-0.183	*2.503×10^-5^
	*TGFB1*	-0.164	*1.714×10^-4^

*: *P*<0.05; TAM: tumor-associated macrophage; Th: T helper cell; Tfh: Follicular helper T cell; Treg: regulatory T cell; CSF1R: Colony Stimulating Factor 1 Receptor; CCL2: C-C Motif Chemokine Ligand 2, IL10: Interleukin 10; INOS (NOS2): Nitric Oxide Synthase 2; IRF5: Interferon Regulatory Factor 5; PTGS2: Prostaglandin-Endoperoxide Synthase 2; VSIG4: V-Set And Immunoglobulin Domain Containing 4; MS4A4A: Membrane Spanning 4-Domains A4A; CEACAM8: Carcinoembryonic Antigen Related Cell Adhesion Molecule 8; ITGAM: Integrin Subunit Alpha M; CCR7: C-C Motif Chemokine Receptor 7; KIR2DL1: Killer Cell Immunoglobulin Like Receptor, Two Ig Domains And Long Cytoplasmic Tail 1; HLA-DPB1: Major Histocompatibility Complex, Class II, DP Beta 1; CD1C: CD1c Molecule; ITGAX: Integrin Subunit Alpha X; NRP1: Neuropilin 1; TBX21: T-Box 21; STAT4: Signal Transducer And Activator Of Transcription 4; IFN-g: Interferon Gamma; TNF-a: Tumor Necrosis Factor; GATA3: GATA Binding Protein 3; BCL6: BCL6 Transcription Repressor; FOXP3: Forkhead Box P3; CCR8: C-C Motif Chemokine Receptor 8; STAT5B: Signal Transducer And Activator Of Transcription 5B; TGFB1:Transforming Growth Factor Beta 1.

### Prognostic analysis of FANCE on pan-cancers

Since FANCE was found to be closely related to the infiltration of a variety of cells in the immune microenvironment, we are interested in whether changes in FANCE expression can predict the prognosis of other cancers.

**Figure 15:**
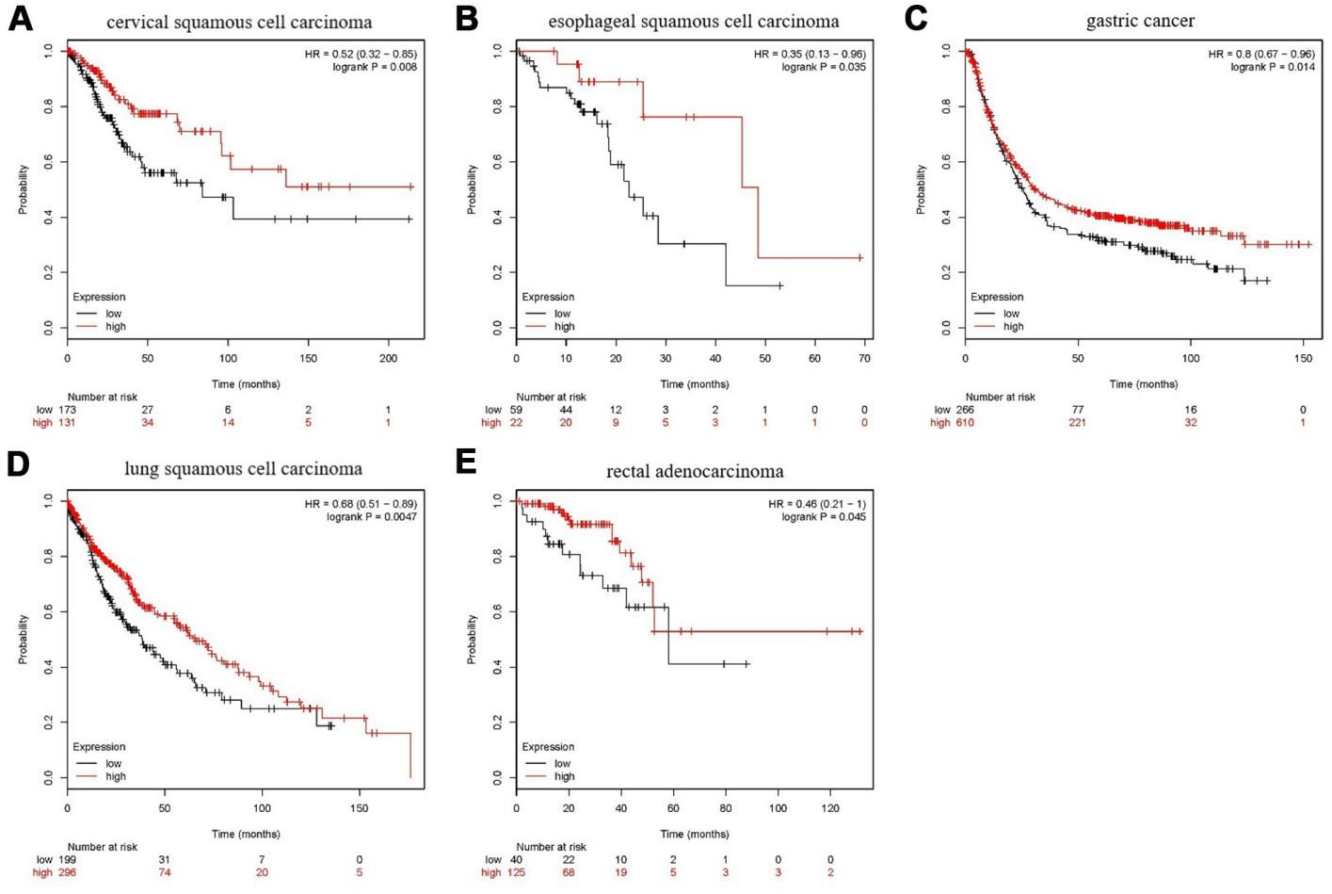
**Prognostic analysis of FANCE in multiple cancers.** (**A**) For cervical squamous cell carcinoma, high expression of FANCE is associated with good prognosis (*P<0.001). (**B**) In esophageal squamous cell carcinoma patients, up-regulated expression of FANCE is associated with good prognosis (*P=0.035); (**C**) For gastric cancer, increased expression of FANCE is associated with better prognosis (*P=0.014); (**D**) For lung squamous cell carcinoma, up-regulated expression of FANCE predicts improved prognosis (*P=4.7×10^-3^); (**E**) For rectal adenocarcinoma, increased expression of FANCE is associated with better prognosis (*P=0.045).

As shown in Figure 15, in five types of tumors: cervical squamous cell carcinoma, esophageal squamous cell carcinoma, gastric cancer, lung squamous cell carcinoma, and rectal adenocarcinoma, high expression of FANCE predicts a good prognosis. The expression of FANCE and macrophage infiltration in these types of tumors were also significantly negatively correlated, suggesting that FANCE may have the same mechanism as HNSC in pan-cancer.

Additionally, the up-regulation of FANCE expression was significantly related to the 5-year survival improvement of patients with thyroid cancer, thymoma, and ovarian cancer. However, in uterine corpus endometrial carcinoma, sarcoma, pheochromocytoma and paraganglioma, high expression of FANCE is significantly associated with poor prognosis.

## DISCUSSION

In this study, 292 signature genes of macrophages were comprehensive analysis in patients with HNSC, and a prognostic model was constructed by random forest model of machine learning algorithm. Five genes were screened out, and FANCE with the highest score was further integrated analyzed. We found that FANCE was highly expressed in HNSC, and hypomethylation of specific methylation sites could be an important reason for the up-regulation. DNA mismatch repair was the function of FANCE that has been revealed, and this study also found that it was related to immune functions such as immunoglobulin binding, monocyte chemotaxis and regulation of macrophage cytokine through GSEA. The expression of FANCE significantly suppressed the infiltration levels of CD4+ T cells, neutrophils, macrophages, and DCs, as well as the marker genes of subclasses of CD4+ T cells and M2 macrophage. These results suggest that FANCE may inhabit lymphocytes and macrophages in the immune microenvironment. Finally, the results of pan-cancer analysis showed that the transcriptional expression of FANCE can predict favorable prognosis of various malignancies including HNSC.

Random forest is an integrated learning method for classification, regression and other tasks [24]. It functions through a large number of decision trees during training and further outputs classes as a classification or average prediction model of individual trees [25]. One of the important advantages of random forest is that it can estimate the feature importance of variables. The random forest model provides two methods for evaluating the importance of variables. “Mean decrease Gini (MDG)” calculates the influence of each variable on the heterogeneity of the observed value at each node of the classification tree. A higher MDG indicates that the degree of impurity arising from the category could be reduced farthest by one variable and thus suggests an important associated index [26]. Another indicator “mean decrease accuracy (MDA)” indicates the degree of decrease in the accuracy of random forest prediction after randomly replacing a variable [27]. It reflects the predicted strength of the variable and represents the importance of the variable. In this study, we used two methods respectively and achieved the same results. Previous researchers [28, 29] mostly identified hub genes in HNSC by establishing co-expression networks or PPI networks, but in this study, we applied a combination of different methods to better ensure the accuracy of the screened hub genes. The constructed five-gene model can accurately predict the prognosis of HNSC.

FANCE acquired highest feature importance score, which mean that it contributed the most to the prognosis in the random forest model. However, this definitely does not mean that FANCE is the most pivotal, critical or indispensable signature gene set of macrophages. Actually, the hub genes have been shown to be associated with different malignancy. UTP3 was proved to be involved in the pathogenesis of colorectal cancer [30] and ADAMDEC1 was a positive regulator of epithelial defense against cancer [31] and plays an active role in inhibiting glioblastoma development [32]. DNAJC13 promoted breast cancer progression [33], and the overexpression of DNASE1L3 was a good prognostic index for liver cancer and renal cancer [34]. FANCE was selected as the research object for further analysis based on the following considerations: among the five hub genes, one study [35] has preliminarily confirmed that FANCE was related to the prognosis of HNSC, but public studies of the other four genes and HNSC were not detected. Fanconi anemia (FA) is a rare autosomal recessive chromosome breakage disease characterized by aplastic anemia in childhood, susceptibility to cancer and cellular sensitivity to DNA cross-linking [36]. The FA protein encoded by FANCE and other cloned FA genes (FANCA, FANCC, FANCD2, FANCF and FANCG) cooperates in a common way, resulting in the homogenization of FANCD2 protein and the coexistence of FANCD2 and breast cancer type 1 susceptibility protein (BRCA1) in nuclear lesions [37]. These BRCA1 sites are involved in the process of DNA repair mediated by homologous recombination. Novel evidence demonstrated that mutation of FA genes predisposed to development of different cancers. Single nucleotide polymorphisms of FANCE in the DNA repair pathways have been associated with increased risk of esophageal cancer [38]. The study by Bonache showed that FANCE was included in the gene set of 18 genes with loss-of-function variants in breast or ovarian cancer patients, three of which also carried pathogenic variants in known cancer genes [39]. Similarly, another study of genome sequencing of 66 patients with sarcoma also found that one patient had FANCE nonsense mutation [40]. An increased mutation load of variants in FANCE in HNSC patients was observed compared with population-level estimate by Chandrasekharappa [35]. In the above studies, the proportion of FANCE mutation was low and it was only one of multiple mutant genes. Other members of FA family had also been frequently detected mutations. These findings can be interpreted as the lack of double-stranded DNA repair mediated by homologous recombination in many cancers, including HNSC, because FA gene knockout experiments have shown that only one FA gene inactivation was enough to destroy the entire FA pathway. Disruption of the FA pathway can explain the large chromosomal changes that are common in sporadic cancers. The present study suggested that HNSC is frequently marked by transcriptional up-regulation of FANCE, which is different from Wreesmann's research. Wreesmann detected the FA gene expression in 49 tongue cancer specimens and found that downregulation of FANCE in sporadic HNSC was rare [41]. This is due to the different sources of tissue. Both oral cancer and oropharyngeal cancer were included in the present study, and the expression of FANCE in oropharyngeal tissues was significantly higher than that in oral tissues as shown in Table 1. We further explored the underlying causes of the observed transcriptional changes and focused on the frequency of methylation changes. The down-regulation of gene expression caused by hypermethylation of CpG islands in gene promoter regions is a common gene silencing mechanism in epigenetic regulation. Among the 10 methylation sites of FANCE, cg12798052 located in the promoter region showed significant hypomethylation in HNSC patients, which is an important reason for the down-regulation of FANCE expression. In addition, the methylation sites cg03030757, cg18744234 and cg15267307 located in the gene enhanced region were also found to be hypomethylated. The DNA modification on different structural elements such as the promoter, coding region or distal enhancer region of the gene, as well as the combined action of transcription factors and microRNA, constitute a complex regulatory system for gene transcription. Whether other epigenetic modifications, such as histone deacetylation or chromatin remodeling, affect the expression or function of FANCE requires further study.

Fanconi anaemia proteins are known to be involved in cell cycle regulation. In FA it is possible to observe accumulation of cells in the G2/M phase of the cell cycle. Sala-Trepat [42] showed that in contrast to normal cells, FA cells could be inefficient in arresting S phase cell cycle progression in response to lesions induced by crosslinking agents. Moreover, various studies [43–45] provide accumulating evidence that FA proteins participate in cell apoptosis. It has been generally observed that FA lymphoblasts spontaneously enter apoptosis *in vitro* more frequently than normal cells [46]. As shown in Table 2, FANCE and Wnt-target pathways are significantly positively correlated, and Wnt / β-catenin pathway is one of the key signal transduction pathways that regulate cell cycle progression and apoptosis. The result suggested that FANCE affected cell cycle and apoptosis through Wnt / β-catenin pathways. Other biological processes such as EMT, angiogenesis, or pan-fibroblast TGF-β response signature, are closely related to the occurrence and development of HNSC, but the relationship between them and FANCE expression is rarely studied. A role of cytochrome P450 enzymes in chromosomal instability of FA primary fibroblasts has been suggested [47]. Electrophoretic mobility shift assays and siRNA experiments confirmed the shared regulatory responses between the prominent members of the TGF-β and JAK/STAT pathways and members of the FA core complex [48].

In FA, the inefficiently constrained innate immune response very likely contributes to the onset and progression of bone marrow failure and clonal selection. Previous study [49] reported that neutrophils, total absolute lymphocytes, B cells, and NK cells decreased, and serum IgG and IgM levels decreased significantly in FA patients. Yujin Sekinaka found that FANC plays an important role in the differentiation of hematopoietic stem cells, and abnormal lymphocyte production is found in most patients with FA [50]. However, the effect of FA gene expression changes on lymphocytes is not yet clear. This study shows that in HNSC patients, the up-regulation of FANCE expression is negatively correlated with lymphocyte infiltration. Analysis of CD4+T cell subclasses showed that Treg, Th1, Th2 and Th17 were significantly down-regulated in the group with high expression of FANCE. The down-regulation of these cells subclasses further suppress the enrichment of cytotoxic T cells, B cells, neutrophils, monocytes / macrophages and dendritic cells. Our results show that there is a negative correlation between FANCE and almost all recognized immune cells, and negative correlations have been observed not only in HNSC but also in various cancers. Tumors of different cancer types may share underlying similarities. Thus, pancancer analysis of large-scale data across a broad range of cancers has the potential to improve disease modeling by exploiting these pancancer similarities. FANCE is derived from xCell algorithm macrophage gene signature, and the included data sources by xCell algorithm also include different types of cells. An association with malignancy in a broad range of tissue types would indicate that FANCE has the potential to be a pan-cancerous biomarker. As shown in Table 3, similar to HNSC, FANCE was negative correlated with macrophage infiltration in 15 other cancers, indicating that FANCE has the same immunoregulatory mechanism in a variety of cancers. This unclear mechanism needs further research to reveal.

Co-expressed genes refer to a set of genes whose expression levels have a trend of synergistic change. These genes are usually synergistic in function and may be regulated by the same transcription mechanism or participate in the same metabolic pathway. Genes that exist in the same pathway generally show a tendency to co-expression, and this feature allows gene function detection. Checkpoint inhibitors, which are monoclonal antibodies, block inhibitory checkpoint antigens and inhibit T cell stimulation, showing anticancer activity [17, 18]. There is evidence that T cells lose their effector function and ability to kill tumor cells when stimulated by tumor antigens, which may be due to the increasing diversity and number of inhibitory receptors, including CD274, PDCD1LG2, HAVCR2, CTLA4, LAG3, PDCD1, TIGIT [21, 23, 51–53]. Our results show that there is a significant co-expression between FANCE and most of these immune checkpoint genes, suggesting that FANCE may be involved in certain mechanisms of immunosuppression. This also confirms that the expression of FANCE has a significant negative correlation with the enrichment of cytotoxic T cells. At present, little is known about whether there is a synergistic function between FANCE expression and immune checkpoint-related genes. Further research is required to reveal the underlying mechanism.

Macrophages are the most abundant immune cells in tumor microenvironment. Much evidences suggest a positive correlation between the extent of TAM infiltration and angiogenesis in tumor tissue [54–56]. Macrophages are mainly differentiated into two distinct phenotypes, including M1 (classical activation) and M2 (alternative activation). M1 macrophages could elaborate the production of pro-inflammatory cytokines, presenting antigen and promoting tumor lysis, while M2 polarized macrophages exhibit inflammatory cytokines, stimulate angiogenesis and promote tumor migration and invasion. The role of typical DNA damage pathways in the phenotype of macrophages is still uncertain. Some studies believe that all hematopoietic defects in FA are only downstream effects of accumulation of DNA damage [57, 58]. However, Garbati's research suggests that abnormal FA protein function can lead to the pro-inflammatory state of macrophages and induce hematopoietic stem cell depletion [59]. Our results preliminarily showed that the up-regulation of FANCE was accompanied by a significant down-regulation of TAM and M2 marker genes, but the M1 marker gene showed a trend of simultaneous up-regulation. If FANCE is further proven to promote TAM depletion or repolarize TAM to M1 macrophages, then FANCE will be attractive as a treatment option. Further research is needed to clarify the role of FANCE in the regulatory network of macrophages.

Different studies have found that FANCE mutations in breast cancer [39], sarcoma [40], colorectal cancer [60], gastric cancer [38] and esophageal cancer [61], but its impact on the prognosis of these malignancies has not been reported. In the current study, we found that in cervical squamous cell carcinoma, esophageal squamous cell carcinoma, gastric cancer, lung squamous cell carcinoma, and rectal adenocarcinoma, the up-regulation of FANCE was associated with both a decrease in macrophage enrichment and a better prognosis. FANCE was responsible for the recruitment of FANCD2, which is a key step in the FA DNA repair pathway. Mutations in FANCE cause damage to the FA pathway, thereby increasing cancer susceptibility. In this study, the overexpression of FANCE is associated with the improved prognosis of a variety of cancers, which is consistent with the results of previous studies [62, 63] and is due to the same biological mechanism. However, in different tumor types, FANCE showed completely opposite effects on prognosis, which suggests the intricate biological mechanism of the FA pathway.

Further research is needed to explore the role of FANCE in HNSC cells and its molecular mechanism that affects different phenotypes of macrophages in tumor microenvironment.

In summary, we use machine learning methods to screen core genes and comprehensively analyze the expression, function, and impact of FANCE on the immune microenvironment in HNSC patients. The present study broadens the existing research, most of which focus on the role of FANCE in Fanconi anemia. Our results provide insight into the potential function of FANCE in tumor immunology and its potential as a biomarker for cancer.

## MATERIALS AND METHODS

### Patients and tissue samples

Oral squamous cell cancer and adjacent normal tissues were collected from patients with HNSC at the Peking University Shenzhen Hospital (Shenzhen, China) between 2018 and 2019. The specimens were all taken from the HNSC resection process, and the distance between tumor tissue and adjacent normal tissue was greater than 2cm. HNSC was diagnosed and classified through pathological examination based on the World Health Organization classification system. Specimens from patients with a history of preoperative chemotherapy were excluded. The study protocol was approved by the Ethics Committees for Human Experiments of Peking University Shenzhen Hospital. All patients signed an informed consent form before sample collection.

### Image processing

The paraffin-embedded tumor sections with the thickness of 5 μm were stained with H&E or antibodies against FANCE (antibody (ab105023) at 1:75 dilution, Abcam Company) according to the routine immunohistochemical staining method. All images shown are wide-field light microscopy images that were acquired at sufficient resolution.

### RNA extraction and qRT-PCR

RNA extraction and qRT-PCR were performed as previously described. Total RNA from frozen tissues or cultured cells was extracted using TRIzol reagent (Invitrogen), according to the manufacturer's protocol. A PrimeScript RT Reagent Kit (Takara Bio, Nojihigashi, Kusatsu, Japan) was used for reverse-transcribing the RNA into cDNA, as per the manufacturer's instructions. qRT-PCR was performed with SYBR-Green Premix Ex Taq (Takara Bio) and was monitored using an ABI PRISM 7500 Sequence Detection System (Applied Biosystems, Life Technologies). Comparative quantification was performed with either the ΔCt or the 2^–ΔΔCt^ method.

### Macrophage-associated genes

Macrophage-associated genes have been compiled in the previous literature [16], and the gene list consists of 292 genes (Supplementary Table 1).

### Acquisition of mRNA data

The gene expression and methylation data, as well as the corresponding clinical information, was downloaded from the cancer genome atlas (TCGA) website (https://portal.gdc.cancer.gov) for HNSC and estimated as log2 (x+1) transformed RSEM normalized counts. All data were processed using R-studio software (version 3.5.3).

### Network analysis databases for protein–protein interaction network analysis database

The search tool for the retrieval of interacting genes/proteins (STRING) database (https://string-db.org/) was used to predict the association between hub gene in regulatory network analysis. PPI node pairs with a combined score ≥ 0.4 were selected for further analysis. The hub genes in the PPI network were identified according to degree using Cytoscape software (version 3.6.1). ClueGO app was used for gene ontology (GO) and KEGG enrichment analysis.

### Target genes identification

We firstly used the "survival" package in R software to perform univariate Cox regression analysis to initially screen out the genes that affect the prognosis. Survival curves with mortality hazard ratios (HRs) were generated using the Kaplan–Meier method and the differences between survival curves were calculated using a log-rank test. The Wald test was used and *P*<0.05 was considered to indicate a statistically significant difference.

The genes screened out with significant prognosis were next included for randomForest feature selection using “RandomForest” package. Mtry is set to the square root of the maximum number of variables, and ntree is set to 1000 in the model. The minimum number of samples required to split a node was set to two. The minimum samples per leaf was set to one. A prognostic model was constructed by random forest algorithm to screen the genes which reached the standards (both Mean Decrease Gini and Mean Decrease Accuracy ranked top 5) were considered as candidate hub genes.

### Validation of the prognostic model

The five genes screened out from the random forest model were identified as the candidate risk factors. Then, by weighting expression value of each gene to corresponding regression coefficients in the univariate COX regression analysis, we established the risk score of each gene respectively. Patients with HNSC were equally stratified into low and high-risk subtypes according to the median cut-off risk score separately. Then, the differences in prognosis between the two groups were compared, and the predictive ability of the model was assessed by receiver operating characteristic curve (ROC) and area under the curve (AUC). The risk scores of all patients were ranked in order from high to low, and then we used each risk score as a threshold to divide the samples into two groups. Each time the threshold was taken, the false positive rate (FPR) and true positive rate (TPR) could be calculated and marked as a point on the coordinate. The ROC curve was established by connecting all points on the coordinate, and the AUC was calculated by the survivalROC R package.

### Expression of hub gene in HNSC

The hub gene with the highest score in the random forest model was chosen for further analysis. Expression levels of hub genes were compared between tumors and normal samples, as well as paired samples. The t test was used to detect whether the difference was statistically significant. Image-based immunohistochemistry protein data for normal and cancer samples are available in the human protein atlas (https://www.proteinatlas.org/).

### Methylation and gene expression analyses

DNA methylation data from TCGA contains β values for 485,577 CpG sites. The β value is calculated as (M / M + U) and ranges from 0 to 1, where M is the frequency of the methylated allele and U is the frequency of the unmethylated allele. Therefore, higher β values indicate higher levels of methylation. The levels of hub gene methylation between HNSC and normal tissues were compared. In addition, we investigated the association between hub gene expression and its DNA methylation status.

### Gene set enrichment analysis and single sample gene set enrichment

To identify its potential biological mechanism, a gene set enrichment analysis (GSEA) was conducted to detect whether a priori defined set of genes showed statistically significant differential expression. Firstly, the FANCE high expression group is selected based on the median expression of FANCE, and the genes are sorted according to expression differences to form a gene list. The annotated gene sets C2.CP (186 gene sets) and C5.BP (5910 gene sets) MSigDB datasets from the Broad Institute were selected as the reference gene sets. These preset gene sets represent different biological processes or signal pathways. Then the GSEA algorithm can determine whether the members of this reference gene set are randomly distributed in the FANCE high expression group gene list, or are mainly enriched at the top or bottom. The third step is to calculate the enrichment score (ES) of the gene set and perform a permutation test of significance to obtain the p value and the false discovery rate (FDR). FDR < 25% and *P* < 0.05 were considered the cut-off criteria. According to the pathway suggested in GSEA, using the GSVA package in R studio software, the correlation coefficient between the target gene and the key pathway can be calculated through a single sample of GSEA (ssGSEA).

### TIMER database analysis

TIMER is a comprehensive resource for the systematic analysis of immune infiltration across diverse types of cancer (https://cistrome.shinyapps.io/timer/). We analyzed the level of the hub gene expression in all available types of cancers and the correlation of its expression with the abundance of immune infiltrates, including B cells, CD4+ T cells, CD8+ T cells, neutrophils, macrophages, and dendritic cells, via gene modules. The immune cell infiltration score of each patient in the TCGA database was obtained from TIMER and was divided into a high score group and a low score group based on the median value.

### Immunological analysis by ESTIMATE R package

The “ESTIMATE” R package was used to predict the presence of infiltrating stromal/immune cells in tumor tissues using gene expression data [64]. It provides an abundance of data for 33 types of lymphocytes, including activated CD8+ cells (Act CD8), central memory CD8 cells (Tcm CD8), effector memory CD8 cells (Tem CD8), activated CD4+ cells (Act CD4), central memory CD4 cells (Tcm CD4), effector memory CD4 cells (Tem CD4), T follicular helper cells (Tfh), gamma delta T cells (Tgd), type 1 T helper cells (Th1), type 17 T helper cells (Th17), type 2 T helper cells (Th2), regulatory T cells (Treg), activated B cells (Act B), immature B cells (Imm B), memory B cells (Mem B), natural killer (NK) cells, CD56 bright NK cells (CD56bright), CD56 dim NK cells (CD56dim), myeloid derived suppressor cells (MDSCs), NK T cells (NKT), activated dendritic cell (Act DCs), plasmacytoid DCs (pDCs), immature DCs (iDCs), major histocompatibility complex II (MHC II), lymphocyte-specific protein tyrosine kinase (LCK), signal transducer and activator of transcription 1 (STAT1), macrophages, eosinophils, mast cells (Mast), monocytes, interferon, and neutrophils in different types of cancers. The relationship between the abundance of tumor-infiltrating lymphocytes and the hub gene expression was analyzed in R studio.

### Prognostic analysis of pan-cancers

The Kaplan Meier plotter (KM plotter, http://kmplot.com/analysis/) is capable of assessing the effect of 54,000 genes on survival in 21 types of cancer [65]. The correlation between the hub gene expression and survivals of all 21 types of cancers were analyzed by Kaplan-Meier plotter. The hazard ratio (HR) with 95% confidence intervals (CI) and log-rank P value were also computed.

### Co-expression analysis in cBioPortal

The cBioPortal for cancer genomics (https://www.cbioportal.org) is an open-access, open-source resource for the interactive exploration of multidimensional cancer genomics data sets [66, 67]. The correlation between the hub gene expression and gene markers of immune cells were explored. The gene markers of the immune cells included markers of CD8+ T cells, T cells (general), B cells, monocytes, TAMs, M1 macrophages, M2 macrophages, neutrophils, NK cells, DCs, Th1 cells, Th2 cells, follicular helper T (Tfh) cells, Th17 cells, Tregs, and exhausted T cells. These gene markers are referenced in prior studies. The Spearman method was used to identify the correlation coefficient.

### Statistical analysis

For the univariate COX analysis, we use Wald test to evaluate whether genes have a significant effect on the prognosis. For survival analysis, Kaplan–Meier method and the differences between survival curves were calculated using a log-rank test. The t test was used to analyze the difference in gene expression between HNSC and the control group, and logistic regression was used to analyze the expression of FANCE Association with clinicopathological variables. In the GSEA analysis, we use the permutation test to obtain the p-value, and this process is automatically calculated in the software. We use Spearman coefficients to evaluate the correlation between FANCE and the other genes, and t-test is used to evaluate statistical significance. P values < 0.05 were statistically significant.

## Supplementary Material

Supplementary Table 1

Supplementary Table 2

Supplementary Table 3

Supplementary Tables 4 and 5

Supplementary Table 6

Supplementary Table 7
