# Supplementary material for: Comprehensive analysis of macrophage-related multigene signature in the tumor microenvironment of head and neck squamous cancer
Source: Aging (Albany NY). 2021 Feb 11;13(4):5718–47. doi: 10.18632/aging.202499 (PMC7950226; doi:10.18632/aging.202499)
Supplement: Supplementary Table 1 [file aging-13-202499-s001.docx]

**Supplementary Table 1. Gene marker for macrophage^*^.**

| **Gene Symbol** |
| --- |
| ACP2 |
| CLCN7 |
| ADRA2B |
| ABCD1 |
| ADCY3 |
| FGR |
| ALK |
| AP1B1 |
| DNASE1L3 |
| CD52 |
| FDX1 |
| ALCAM |
| GLB1 |
| ARSB |
| ALDH9A1 |
| GPD1 |
| CCL8 |
| TSPO |
| HEXA |
| ATP6V0A1 |
| ANXA11 |
| MSR1 |
| CCL22 |
| ATOX1 |
| C3AR1 |
| HEXB |
| CD63 |
| AQP8 |
| GUCA1A |
| NPR1 |
| CD163 |
| ATP6V0C |
| DAGLA |
| C1QA |
| ANXA2 |
| ATP2A2 |
| HS3ST2 |
| KCNJ1 |
| UCP3 |
| ADAMDEC1 |
| ATP6V1E1 |
| CALR |
| C1QB |
| ATP6V1A |
| ATP6V1C1 |
| FKBP15 |
| TPP1 |
| ELK1 |
| TREM2 |
| BLVRA |
| CHIT1 |
| PQLC2 |
| SLC31A1 |
| FH |
| PDE1B |
| MYO15A |
| HAMP |
| CYBB |
| TMEM70 |
| FLT1 |
| CD48 |
| CYC1 |
| CAMP |
| SLC38A7 |
| CYP19A1 |
| CANX |
| HADHB |
| NCKAP1L |
| SDS |
| DLAT |
| CMKLR1 |
| CD80 |
| MS4A4A |
| FCER1G |
| CD81 |
| CLTC |
| GP1BA |
| CSF1 |
| LAMP1 |
| DNASE2B |
| CCR1 |
| CSF1R |
| IFNAR1 |
| MMP19 |
| MYOZ1 |
| KCNJ5 |
| IL10 |
| MYO9B |
| NDUFB1 |
| COX5B |
| P2RX7 |
| KIFC3 |
| FANCE |
| FTL |
| SDCBP |
| MT2A |
| MARCO |
| PEX19 |
| FOLR2 |
| MYBPH |
| ATP6V0D1 |
| S100A6 |
| FPR3 |
| MYH11 |
| SLC6A7 |
| MYO7A |
| PDCD6IP |
| SLC6A12 |
| FPR2 |
| COL4A3BP |
| SNAPC2 |
| HK3 |
| PRDX1 |
| HPS1 |
| CYFIP1 |
| SNX1 |
| RAB3IL1 |
| PLEKHM2 |
| TAF10 |
| IL12B |
| RNH1 |
| ITGAX |
| ITGAE |
| MRPL12 |
| SMG5 |
| UGP2 |
| LAIR1 |
| CCL1 |
| SLC39A1 |
| USF2 |
| CXCL9 |
| CCL7 |
| COMMD9 |
| XPNPEP2 |
| AKR7A2 |
| NARS |
| CCL24 |
| STX18 |
| SNX3 |
| NDUFS2 |
| SRC |
| TNFSF14 |
| VIM |
| NFS1 |
| PDCL |
| RRP1 |
| GSTO1 |
| MAPK13 |
| SNX2 |
| VPS53 |
| PTGIR |
| S1PR2 |
| S100A11 |
| STX4 |
| PTPRA |
| AP1M2 |
| LONRF3 |
| BCAP31 |
| RELA |
| ACTR3 |
| CDS2 |
| CEPT1 |
| LILRB1 |
| PABPC4 |
| BAIAP2 |
| AFG3L2 |
| CCL18 |
| SLC9A6 |
| CCL19 |
| PICK1 |
| VTI1B |
| LILRB4 |
| ARHGEF11 |
| ARFGEF2 |
| EMILIN1 |
| HSPH1 |
| VSIG4 |
| SLC1A2 |
| LILRA2 |
| HSPB7 |
| SCAMP2 |
| EFR3A |
| TFRC |
| COQ2 |
| SLC11A1 |
| AGPS |
| SPR |
| SIGLEC1 |
| NCAPH |
| OS9 |
| ZCCHC4 |
| WSB2 |
| TIE1 |
| TMED5 |
| CD84 |
| SLAMF8 |
| USP14 |
| PKD2L1 |
| HYAL2 |
| NAGPA |
| ITGB1BP1 |
| CLPB |
| GABARAP |
| ZNF219 |
| ATP6V1F |
| LONP1 |
| TFEC |
| TRIP4 |
| ADCK2 |
| IGSF6 |
| WDFY3 |
| ANGPT4 |
| TBC1D9B |
| ATP6V1D |
| CIAO1 |
| CD300C |
| ZC3H3 |
| WTAP |
| KCTD5 |
| ABI1 |
| AGGF1 |
| MFN1 |
| ACTR2 |
| PHLDB1 |
| UNC50 |
| IARS2 |
| GGA1 |
| CCDC88A |
| ZMPSTE24 |
| CLEC4E |
| SNX5 |
| KCNK13 |
| BCKDK |
| MYOF |
| TMEM9B |
| EXOC5 |
| POGK |
| STIP1 |
| UQCR11 |
| GLRX2 |
| SLC25A24 |
| IPPK |
| SPG21 |
| CARD14 |
| ATP6V1H |
| ALG9 |
| OGFR |
| ANKFY1 |
| MRM1 |
| NDUFAF1 |
| BTBD1 |
| DHX57 |
| TMEM33 |
| SLC25A46 |
| DNAJC13 |
| RIN2 |
| OSBPL11 |
| TDRD7 |
| CCDC85C |
| STX12 |
| IL17RA |
| ACSM5 |
| ABTB2 |
| FAM32A |
| SIGLEC7 |
| PLEKHB2 |
| ADO |
| SIGLEC9 |
| NOP10 |
| TBC1D16 |
| VPS35 |
| NRBP1 |
| TMEM184C |
| EXOC1 |
| OTUD4 |
| HAUS2 |
| ARL8B |
| NECAP2 |
| MTMR14 |
| WDR11 |
| GORASP1 |
| ZC3H15 |
| CCDC47 |
| UTP3 |
| UBXN6 |
| MRS2 |
| MRPL40 |
| VPS33A |
| CORO7 |
| LIMD2 |
| TMX1 |
| DOT1L |

*The gene list is base on Aran D, Hu Z, Butte AJ: xCell: digitally portraying the tissue cellular heterogeneity landscape. Genome biology 2017;18:220.
