# Supplementary material for: Comprehensive analysis of macrophage-related multigene signature in the tumor microenvironment of head and neck squamous cancer
Source: Aging (Albany NY). 2021 Feb 11;13(4):5718–47. doi: 10.18632/aging.202499 (PMC7950226; doi:10.18632/aging.202499)
Supplement: Supplementary Table 2 [file aging-13-202499-s002.pdf]

SUPPLEMENTARY TABLE

Supplementary Table 2. Analysis of macrophage markers genes by univariate COX.

Gene	HR	HR.95L	HR.95H	P-value
CYP19A1	4.522	2.342	8.733	$7.000*10^{-6}$
LIMD2	0.912	0.871	0.955	$8.030*10^{-5}$
MYO9B	0.944	0.914	0.975	$5.717*10^{-4}$
DOT1L	0.885	0.822	0.953	$1.202*10^{-3}$
CD52	0.986	0.977	0.995	$1.462*10^{-3}$
CCL1	1.407	1.128	1.754	$2.407*10^{-3}$
CD48	0.938	0.900	0.978	$2.535*10^{-3}$
BCAP31	1.003	1.001	1.005	$6.353*10^{-3}$
NCKAP1L	0.914	0.856	0.976	$6.977*10^{-3}$
VPS35	1.037	1.010	1.066	$7.119*10^{-3}$
MMP19	0.938	0.894	0.984	$9.268*10^{-3}$
CLCN7	0.933	0.886	0.983	$9.412*10^{-3}$
FDX1	1.078	1.018	1.140	$9.683*10^{-3}$
UTP3	1.028	1.006	1.051	$1.257*10^{-2}$
TMEM9B	1.030	1.006	1.054	$1.369*10^{-2}$
IL10	0.622	0.420	0.921	$1.761*10^{-2}$
FANCE	0.975	0.954	0.996	$2.078*10^{-2}$
ANXA2	1.002	1.000	1.003	$2.096*10^{-2}$
S1PR2	0.900	0.822	0.984	$2.128*10^{-2}$
SDCBP	1.014	1.002	1.026	$2.437*10^{-2}$
MYH11	1.013	1.002	1.025	$2.625*10^{-2}$
SLC6A7	0.013	0.000	0.606	$2.669*10^{-2}$
AGPS	1.035	1.004	1.068	$2.672*10^{-2}$
DNAJC13	0.958	0.922	0.995	$2.702*10^{-2}$
CD84	0.881	0.788	0.986	$2.781*10^{-2}$
STIP1	1.006	1.001	1.011	$3.085*10^{-2}$
MYOF	1.009	1.001	1.017	$3.282*10^{-2}$
IL12B	0.208	0.049	0.886	$3.365*10^{-2}$
NAGPA	0.863	0.752	0.990	$3.483*10^{-2}$
DNASE1L3	1.018	1.001	1.035	$3.803*10^{-2}$
OSBPL11	0.958	0.919	0.998	$4.025*10^{-2}$
SCAMP2	1.016	1.001	1.032	$4.050*10^{-2}$
ADAMDEC1	0.966	0.934	0.999	$4.106*10^{-2}$
DLAT	1.036	1.001	1.073	$4.375*10^{-2}$
UBXN6	0.981	0.963	0.999	$4.409*10^{-2}$
GP1BA	0.749	0.561	1.000	$4.981*10^{-2}$
