# Supplementary material for: Comprehensive analysis of macrophage-related multigene signature in the tumor microenvironment of head and neck squamous cancer
Source: Aging (Albany NY). 2021 Feb 11;13(4):5718–47. doi: 10.18632/aging.202499 (PMC7950226; doi:10.18632/aging.202499)
Supplement: Supplementary Table 3 [file aging-13-202499-s003.docx]

**Supplementary Table 3. Expression of FANCE in normal and HNSC tissue.**

| Patients | Types | Expression of FANCE |
| --- | --- | --- |
| TCGA-CV-7177 | Normal | 6.448477905 |
| TCGA-HD-8635 | Normal | 4.885357822 |
| TCGA-CV-6943 | Normal | 4.338543649 |
| TCGA-CV-7250 | Normal | 4.41484975 |
| TCGA-CV-7438 | Normal | 6.272020205 |
| TCGA-WA-A7GZ | Normal | 4.531920239 |
| TCGA-CV-6959 | Normal | 5.670946031 |
| TCGA-CV-7238 | Normal | 2.669154294 |
| TCGA-CV-7423 | Normal | 4.886702718 |
| TCGA-H7-A6C5 | Normal | 3.625142974 |
| TCGA-CV-7242 | Normal | 4.359282565 |
| TCGA-CV-7440 | Normal | 5.641184068 |
| TCGA-CV-6961 | Normal | 4.013741188 |
| TCGA-CV-7245 | Normal | 2.215915124 |
| TCGA-CV-7416 | Normal | 4.504562016 |
| TCGA-CV-6938 | Normal | 5.641356245 |
| TCGA-CV-7101 | Normal | 8.43816359 |
| TCGA-CV-6960 | Normal | 7.561391089 |
| TCGA-CV-7434 | Normal | 2.242350326 |
| TCGA-CV-7183 | Normal | 6.36133641 |
| TCGA-CV-7252 | Normal | 4.581651943 |
| TCGA-HD-A6HZ | Normal | 3.182422999 |
| TCGA-CV-6934 | Normal | 6.58527099 |
| TCGA-CV-7255 | Normal | 6.993466272 |
| TCGA-HD-A6I0 | Normal | 1.962784244 |
| TCGA-CV-7424 | Normal | 6.478547809 |
| TCGA-CV-7261 | Normal | 11.11876024 |
| TCGA-H7-A6C4 | Normal | 2.469733377 |
| TCGA-CV-7097 | Normal | 2.551280952 |
| TCGA-CV-7091 | Normal | 1.386408086 |
| TCGA-CV-6956 | Normal | 5.715106667 |
| TCGA-CV-6939 | Normal | 1.339942754 |
| TCGA-CV-6962 | Normal | 7.272354399 |
| TCGA-CV-7432 | Normal | 3.016398651 |
| TCGA-CV-7178 | Normal | 5.380158915 |
| TCGA-CV-6955 | Normal | 5.386422691 |
| TCGA-CV-6935 | Normal | 5.652059057 |
| TCGA-CV-6936 | Normal | 4.78916536 |
| TCGA-CV-7425 | Normal | 6.08632076 |
| TCGA-CV-7437 | Normal | 6.584120952 |
| TCGA-CV-7235 | Normal | 2.977478339 |
| TCGA-CV-7406 | Normal | 2.328782586 |
| TCGA-CV-7103 | Normal | 5.686144365 |
| TCGA-CV-6933 | Normal | 1.529861739 |
| TCGA-KU-A6H7 | Normal | 30.9665178 |
| TCGA-UF-A71A | Normal | 17.20650691 |
| TCGA-BA-4077 | HNSC | 12.2481167 |
| TCGA-QK-A8Z9 | HNSC | 11.03711283 |
| TCGA-CV-A6K0 | HNSC | 4.983210072 |
| TCGA-CV-A6JO | HNSC | 4.240117126 |
| TCGA-BA-6868 | HNSC | 15.91694636 |
| TCGA-CQ-A4CA | HNSC | 16.83121566 |
| TCGA-CQ-A4CE | HNSC | 5.492839654 |
| TCGA-QK-A6IF | HNSC | 6.888111661 |
| TCGA-CV-6003 | HNSC | 7.162196498 |
| TCGA-BA-A6DG | HNSC | 6.089687739 |
| TCGA-BA-4075 | HNSC | 13.36574862 |
| TCGA-CN-4741 | HNSC | 20.46539652 |
| TCGA-CR-7390 | HNSC | 12.74698258 |
| TCGA-BA-A6DJ | HNSC | 15.10831801 |
| TCGA-CV-6935 | HNSC | 16.61917616 |
| TCGA-CN-4725 | HNSC | 10.91547551 |
| TCGA-CV-5444 | HNSC | 6.56887924 |
| TCGA-CV-7255 | HNSC | 11.324035 |
| TCGA-CR-6484 | HNSC | 6.127410167 |
| TCGA-CQ-A4CD | HNSC | 12.04422448 |
| TCGA-F7-A61S | HNSC | 5.989317243 |
| TCGA-QK-A6IJ | HNSC | 5.835615259 |
| TCGA-D6-6517 | HNSC | 8.441483589 |
| TCGA-CQ-7065 | HNSC | 8.923799495 |
| TCGA-BB-A6UO | HNSC | 7.331489069 |
| TCGA-CN-A498 | HNSC | 19.97381033 |
| TCGA-CQ-5329 | HNSC | 13.57857031 |
| TCGA-D6-A4ZB | HNSC | 27.36758748 |
| TCGA-CV-7413 | HNSC | 6.893145358 |
| TCGA-CV-6952 | HNSC | 9.472984638 |
| TCGA-CR-7380 | HNSC | 6.914013872 |
| TCGA-CR-7367 | HNSC | 3.646757887 |
| TCGA-IQ-A6SG | HNSC | 17.08813694 |
| TCGA-CQ-5327 | HNSC | 7.297452443 |
| TCGA-CV-6441 | HNSC | 8.50883659 |
| TCGA-BA-6870 | HNSC | 16.55712325 |
| TCGA-MT-A7BN | HNSC | 2.17960133 |
| TCGA-CV-A6JZ | HNSC | 10.35841736 |
| TCGA-CQ-7071 | HNSC | 8.224596281 |
| TCGA-CV-6948 | HNSC | 8.107588805 |
| TCGA-CV-6962 | HNSC | 14.70198091 |
| TCGA-CN-A63T | HNSC | 6.299417202 |
| TCGA-CR-6473 | HNSC | 14.65532234 |
| TCGA-CV-7423 | HNSC | 5.58256426 |
| TCGA-CN-6019 | HNSC | 8.923979803 |
| TCGA-CN-4728 | HNSC | 5.3010791 |
| TCGA-CR-7377 | HNSC | 4.079486815 |
| TCGA-P3-A6T0 | HNSC | 6.41304773 |
| TCGA-CR-7399 | HNSC | 8.166473235 |
| TCGA-CN-5361 | HNSC | 9.935804369 |
| TCGA-F7-8489 | HNSC | 12.32786271 |
| TCGA-BA-A4IF | HNSC | 18.22741351 |
| TCGA-CN-6992 | HNSC | 19.59049012 |
| TCGA-D6-A74Q | HNSC | 8.630087554 |
| TCGA-CV-6959 | HNSC | 12.70210582 |
| TCGA-CN-A497 | HNSC | 10.86889633 |
| TCGA-MZ-A7D7 | HNSC | 6.071738203 |
| TCGA-CQ-A4CI | HNSC | 19.62042878 |
| TCGA-CV-5978 | HNSC | 6.153520342 |
| TCGA-MT-A51X | HNSC | 6.454300079 |
| TCGA-CQ-5325 | HNSC | 5.055978793 |
| TCGA-CV-7263 | HNSC | 11.79675985 |
| TCGA-D6-6826 | HNSC | 11.23842876 |
| TCGA-UF-A7JJ | HNSC | 16.32773021 |
| TCGA-UF-A719 | HNSC | 34.50812716 |
| TCGA-CQ-6227 | HNSC | 6.089205168 |
| TCGA-D6-A6EO | HNSC | 15.98439407 |
| TCGA-CR-7371 | HNSC | 4.591780834 |
| TCGA-CX-7082 | HNSC | 12.50640612 |
| TCGA-CN-A499 | HNSC | 12.15207899 |
| TCGA-D6-6825 | HNSC | 6.883135825 |
| TCGA-WA-A7H4 | HNSC | 9.613465811 |
| TCGA-F7-A620 | HNSC | 7.426197934 |
| TCGA-CV-5436 | HNSC | 5.200865688 |
| TCGA-CR-5250 | HNSC | 18.86285609 |
| TCGA-CN-4727 | HNSC | 17.83603208 |
| TCGA-DQ-5624 | HNSC | 5.683833176 |
| TCGA-CV-5430 | HNSC | 19.06169461 |
| TCGA-CV-7100 | HNSC | 15.64705222 |
| TCGA-CQ-5324 | HNSC | 8.952656134 |
| TCGA-CR-6478 | HNSC | 7.633381717 |
| TCGA-P3-A6SX | HNSC | 19.17112331 |
| TCGA-BA-A6DL | HNSC | 7.46662619 |
| TCGA-BA-A6DB | HNSC | 5.813545429 |
| TCGA-CR-7370 | HNSC | 21.38203366 |
| TCGA-CV-6942 | HNSC | 7.066126788 |
| TCGA-CV-6433 | HNSC | 19.0835338 |
| TCGA-UF-A7JT | HNSC | 7.725685655 |
| TCGA-CQ-A4CH | HNSC | 6.914838854 |
| TCGA-CV-A468 | HNSC | 3.955603232 |
| TCGA-BA-6873 | HNSC | 13.93589722 |
| TCGA-CV-7254 | HNSC | 6.770179065 |
| TCGA-TN-A7HI | HNSC | 8.209318593 |
| TCGA-CV-A463 | HNSC | 8.404816892 |
| TCGA-CV-6436 | HNSC | 2.671752117 |
| TCGA-CR-7391 | HNSC | 4.279684261 |
| TCGA-CR-7379 | HNSC | 4.511428583 |
| TCGA-C9-A480 | HNSC | 7.127205744 |
| TCGA-CR-7393 | HNSC | 3.798612556 |
| TCGA-CV-6960 | HNSC | 7.025494634 |
| TCGA-CV-7097 | HNSC | 10.65048969 |
| TCGA-QK-A6IH | HNSC | 5.661979312 |
| TCGA-CR-6467 | HNSC | 22.22808317 |
| TCGA-HD-7754 | HNSC | 29.15490627 |
| TCGA-H7-A6C4 | HNSC | 6.266125779 |
| TCGA-BA-4078 | HNSC | 24.19466528 |
| TCGA-CV-7414 | HNSC | 8.945306027 |
| TCGA-CN-A6V3 | HNSC | 9.950014913 |
| TCGA-CV-7102 | HNSC | 16.14464406 |
| TCGA-QK-A6V9 | HNSC | 20.85115023 |
| TCGA-HD-8314 | HNSC | 13.56347587 |
| TCGA-CQ-7063 | HNSC | 8.541302399 |
| TCGA-CR-7369 | HNSC | 11.75923924 |
| TCGA-CV-7178 | HNSC | 11.71185774 |
| TCGA-CN-5374 | HNSC | 13.38898038 |
| TCGA-BB-A5HZ | HNSC | 8.141476438 |
| TCGA-BA-5559 | HNSC | 15.9873253 |
| TCGA-CV-6951 | HNSC | 9.844891172 |
| TCGA-BB-4228 | HNSC | 28.82300705 |
| TCGA-CR-7368 | HNSC | 23.94017872 |
| TCGA-HD-A6I0 | HNSC | 7.497811298 |
| TCGA-F7-A50G | HNSC | 11.28340618 |
| TCGA-DQ-5629 | HNSC | 17.05971449 |
| TCGA-C9-A47Z | HNSC | 11.32831784 |
| TCGA-CV-7242 | HNSC | 9.362439224 |
| TCGA-CR-6472 | HNSC | 9.997571233 |
| TCGA-BA-5556 | HNSC | 7.933917404 |
| TCGA-DQ-7591 | HNSC | 16.2824703 |
| TCGA-CR-7386 | HNSC | 5.016077362 |
| TCGA-BB-A5HU | HNSC | 9.049314689 |
| TCGA-D6-A6EP | HNSC | 9.635146349 |
| TCGA-QK-A8Z7 | HNSC | 17.34864051 |
| TCGA-CX-7086 | HNSC | 10.55968899 |
| TCGA-D6-A4Z9 | HNSC | 10.91358448 |
| TCGA-CV-A6JT | HNSC | 3.874992362 |
| TCGA-TN-A7HJ | HNSC | 8.995120771 |
| TCGA-KU-A66T | HNSC | 19.99763488 |
| TCGA-BB-8601 | HNSC | 19.99096823 |
| TCGA-CV-6939 | HNSC | 19.08754488 |
| TCGA-HD-A4C1 | HNSC | 10.17409222 |
| TCGA-P3-A5QE | HNSC | 20.9203445 |
| TCGA-CQ-5326 | HNSC | 10.65350882 |
| TCGA-CN-5366 | HNSC | 7.53615823 |
| TCGA-CN-5358 | HNSC | 7.165042835 |
| TCGA-CV-A6JM | HNSC | 4.814021764 |
| TCGA-BA-6871 | HNSC | 12.46327471 |
| TCGA-CQ-6218 | HNSC | 4.547410886 |
| TCGA-QK-A6VC | HNSC | 7.670970496 |
| TCGA-CQ-6222 | HNSC | 5.116299298 |
| TCGA-CN-6012 | HNSC | 21.63974111 |
| TCGA-CR-6491 | HNSC | 11.92971242 |
| TCGA-CV-7101 | HNSC | 11.00130434 |
| TCGA-P3-A6T3 | HNSC | 14.05006411 |
| TCGA-CR-7365 | HNSC | 10.34403769 |
| TCGA-BA-A6DE | HNSC | 11.50494107 |
| TCGA-IQ-A61J | HNSC | 6.606514987 |
| TCGA-CV-5435 | HNSC | 21.73678975 |
| TCGA-CV-7432 | HNSC | 11.44222552 |
| TCGA-CQ-5332 | HNSC | 12.08133025 |
| TCGA-CR-5248 | HNSC | 17.50676101 |
| TCGA-CN-A6UY | HNSC | 26.11358335 |
| TCGA-KU-A66S | HNSC | 4.674877048 |
| TCGA-CV-A45V | HNSC | 7.855238718 |
| TCGA-CN-6016 | HNSC | 5.95347511 |
| TCGA-UF-A7JO | HNSC | 11.41876192 |
| TCGA-CV-7177 | HNSC | 9.111828696 |
| TCGA-D6-6515 | HNSC | 4.528121154 |
| TCGA-QK-A6IG | HNSC | 4.873895327 |
| TCGA-CV-5443 | HNSC | 10.53563037 |
| TCGA-HD-8635 | HNSC | 7.819542853 |
| TCGA-BB-4223 | HNSC | 26.16681739 |
| TCGA-CV-7434 | HNSC | 7.879035693 |
| TCGA-UF-A71B | HNSC | 10.07886175 |
| TCGA-CQ-A4C6 | HNSC | 6.416683985 |
| TCGA-UF-A7J9 | HNSC | 14.42015132 |
| TCGA-DQ-7592 | HNSC | 12.84780013 |
| TCGA-CV-7438 | HNSC | 8.1586412 |
| TCGA-CN-A63U | HNSC | 12.64505142 |
| TCGA-CV-7415 | HNSC | 16.72120486 |
| TCGA-CQ-6221 | HNSC | 2.581873609 |
| TCGA-CN-6998 | HNSC | 4.511047233 |
| TCGA-MZ-A5BI | HNSC | 6.751602718 |
| TCGA-CN-4740 | HNSC | 6.234641903 |
| TCGA-CV-A45Q | HNSC | 2.823042361 |
| TCGA-QK-A8ZA | HNSC | 30.39275311 |
| TCGA-CV-5979 | HNSC | 8.887158243 |
| TCGA-CN-6997 | HNSC | 4.721646613 |
| TCGA-CN-5367 | HNSC | 16.58924524 |
| TCGA-CN-4734 | HNSC | 7.012383232 |
| TCGA-DQ-7588 | HNSC | 13.89783242 |
| TCGA-D6-A6EK | HNSC | 6.958332467 |
| TCGA-CV-7183 | HNSC | 6.631037981 |
| TCGA-CN-6017 | HNSC | 2.735903664 |
| TCGA-P3-A5Q5 | HNSC | 15.90357841 |
| TCGA-CR-7374 | HNSC | 21.87430992 |
| TCGA-CR-7392 | HNSC | 5.145347021 |
| TCGA-CV-7095 | HNSC | 9.126152767 |
| TCGA-CQ-7069 | HNSC | 3.005483416 |
| TCGA-CN-5364 | HNSC | 10.65646511 |
| TCGA-CN-A63V | HNSC | 3.953132508 |
| TCGA-CV-7238 | HNSC | 4.389969145 |
| TCGA-CN-6021 | HNSC | 14.98674061 |
| TCGA-CN-4723 | HNSC | 14.59922293 |
| TCGA-UF-A7JA | HNSC | 20.63804169 |
| TCGA-CV-5441 | HNSC | 21.17663897 |
| TCGA-P3-A6T2 | HNSC | 9.40944629 |
| TCGA-BA-5153 | HNSC | 23.51222718 |
| TCGA-F7-7848 | HNSC | 18.09265509 |
| TCGA-IQ-7630 | HNSC | 12.10153282 |
| TCGA-CN-6018 | HNSC | 9.010307434 |
| TCGA-HD-7229 | HNSC | 8.228478635 |
| TCGA-CR-6487 | HNSC | 18.71531741 |
| TCGA-CR-7383 | HNSC | 9.230114825 |
| TCGA-CR-5249 | HNSC | 14.88346739 |
| TCGA-P3-A6SW | HNSC | 53.95536894 |
| TCGA-CQ-6223 | HNSC | 8.101968864 |
| TCGA-HD-7831 | HNSC | 6.231885992 |
| TCGA-CR-7364 | HNSC | 10.14021629 |
| TCGA-CV-5442 | HNSC | 14.72270051 |
| TCGA-CV-5432 | HNSC | 12.82347633 |
| TCGA-CV-6953 | HNSC | 6.209780922 |
| TCGA-F7-A622 | HNSC | 7.322547295 |
| TCGA-CV-6956 | HNSC | 20.69060622 |
| TCGA-QK-A8ZB | HNSC | 18.35071877 |
| TCGA-CV-6941 | HNSC | 12.87823543 |
| TCGA-CV-A6JN | HNSC | 5.227298019 |
| TCGA-CN-6995 | HNSC | 14.82104829 |
| TCGA-CV-7236 | HNSC | 4.618470368 |
| TCGA-UF-A7JV | HNSC | 4.123964371 |
| TCGA-P3-A6T8 | HNSC | 12.91712144 |
| TCGA-BA-6869 | HNSC | 15.37050558 |
| TCGA-BA-4076 | HNSC | 21.68796706 |
| TCGA-RS-A6TO | HNSC | 8.536502924 |
| TCGA-CQ-A4CB | HNSC | 10.70609102 |
| TCGA-CR-7395 | HNSC | 8.955922343 |
| TCGA-P3-A6T6 | HNSC | 16.93218993 |
| TCGA-IQ-A61E | HNSC | 15.03643503 |
| TCGA-CN-5360 | HNSC | 17.53200247 |
| TCGA-CQ-7072 | HNSC | 6.799730058 |
| TCGA-CV-6943 | HNSC | 5.036290608 |
| TCGA-CV-7089 | HNSC | 5.36336581 |
| TCGA-D6-A6EN | HNSC | 5.715476636 |
| TCGA-CQ-6225 | HNSC | 12.07337821 |
| TCGA-CV-7411 | HNSC | 8.675939839 |
| TCGA-CV-A45U | HNSC | 17.32014391 |
| TCGA-CV-A45X | HNSC | 6.574196424 |
| TCGA-CV-7430 | HNSC | 5.613283377 |
| TCGA-CV-6940 | HNSC | 10.11686321 |
| TCGA-BB-A6UM | HNSC | 19.00767094 |
| TCGA-CN-6994 | HNSC | 9.317669213 |
| TCGA-CV-A45O | HNSC | 4.783691923 |
| TCGA-QK-A652 | HNSC | 7.035856741 |
| TCGA-CX-A4AQ | HNSC | 12.35403663 |
| TCGA-CR-7401 | HNSC | 5.603225774 |
| TCGA-T3-A92N | HNSC | 6.444069976 |
| TCGA-CV-7407 | HNSC | 14.8804929 |
| TCGA-CN-4722 | HNSC | 6.980740868 |
| TCGA-UF-A7JK | HNSC | 13.057855 |
| TCGA-CR-6471 | HNSC | 13.07514908 |
| TCGA-CV-A45Z | HNSC | 13.78434753 |
| TCGA-CQ-6229 | HNSC | 8.845834554 |
| TCGA-T2-A6WZ | HNSC | 7.296383249 |
| TCGA-P3-A6T4 | HNSC | 4.342866637 |
| TCGA-CV-6945 | HNSC | 13.31777189 |
| TCGA-BB-4227 | HNSC | 18.98350753 |
| TCGA-BA-A4IG | HNSC | 13.24646309 |
| TCGA-UF-A71D | HNSC | 18.43156482 |
| TCGA-IQ-A61I | HNSC | 15.06710952 |
| TCGA-D6-8569 | HNSC | 9.411677056 |
| TCGA-MT-A67F | HNSC | 10.45339716 |
| TCGA-CN-6996 | HNSC | 9.018640424 |
| TCGA-CV-5971 | HNSC | 3.411660785 |
| TCGA-CV-6954 | HNSC | 20.56652768 |
| TCGA-CV-7252 | HNSC | 10.66893562 |
| TCGA-CQ-5330 | HNSC | 6.018399382 |
| TCGA-P3-A6T7 | HNSC | 9.162215903 |
| TCGA-CN-6011 | HNSC | 31.44612127 |
| TCGA-CN-4733 | HNSC | 6.220334139 |
| TCGA-CN-5356 | HNSC | 17.56152317 |
| TCGA-F7-A623 | HNSC | 6.302983533 |
| TCGA-CV-7104 | HNSC | 7.135978138 |
| TCGA-CV-A45T | HNSC | 5.469123798 |
| TCGA-CV-7090 | HNSC | 5.479971822 |
| TCGA-CV-6955 | HNSC | 5.692342856 |
| TCGA-CN-4739 | HNSC | 13.13895227 |
| TCGA-CR-6488 | HNSC | 3.877910545 |
| TCGA-CV-5977 | HNSC | 5.865400824 |
| TCGA-CN-6010 | HNSC | 7.990137443 |
| TCGA-CN-4742 | HNSC | 9.095010765 |
| TCGA-CV-6961 | HNSC | 8.670047656 |
| TCGA-IQ-A61G | HNSC | 22.38923401 |
| TCGA-CN-6020 | HNSC | 10.94309978 |
| TCGA-CV-5431 | HNSC | 7.772442356 |
| TCGA-CV-7248 | HNSC | 5.309982955 |
| TCGA-CV-7429 | HNSC | 12.65424044 |
| TCGA-CV-7253 | HNSC | 11.72328594 |
| TCGA-CV-7421 | HNSC | 10.01499645 |
| TCGA-CN-A641 | HNSC | 25.9788067 |
| TCGA-CN-4726 | HNSC | 10.38836438 |
| TCGA-CN-5373 | HNSC | 7.910521071 |
| TCGA-UF-A7JS | HNSC | 6.454409848 |
| TCGA-CV-A464 | HNSC | 8.527586739 |
| TCGA-UF-A71E | HNSC | 20.20996492 |
| TCGA-CV-6934 | HNSC | 5.345447825 |
| TCGA-QK-AA3J | HNSC | 14.59995298 |
| TCGA-CV-7245 | HNSC | 7.177659298 |
| TCGA-CV-6936 | HNSC | 9.993552436 |
| TCGA-CV-7418 | HNSC | 9.837738491 |
| TCGA-CN-4729 | HNSC | 7.940121264 |
| TCGA-CR-6482 | HNSC | 9.33159688 |
| TCGA-DQ-5631 | HNSC | 5.23845521 |
| TCGA-CN-6024 | HNSC | 11.78026689 |
| TCGA-WA-A7GZ | HNSC | 16.66120059 |
| TCGA-CN-5355 | HNSC | 9.371592615 |
| TCGA-IQ-7631 | HNSC | 3.755213855 |
| TCGA-CN-4735 | HNSC | 7.423377152 |
| TCGA-CX-7085 | HNSC | 5.430523704 |
| TCGA-CV-7568 | HNSC | 18.09697261 |
| TCGA-CV-A45P | HNSC | 6.401443255 |
| TCGA-CV-7410 | HNSC | 8.822362222 |
| TCGA-HD-A633 | HNSC | 7.279542671 |
| TCGA-CV-7099 | HNSC | 6.782190219 |
| TCGA-CV-7428 | HNSC | 16.48903198 |
| TCGA-BA-A6DD | HNSC | 11.57486869 |
| TCGA-BA-A6DA | HNSC | 10.00150514 |
| TCGA-CR-6493 | HNSC | 9.802103582 |
| TCGA-CQ-5334 | HNSC | 10.32747649 |
| TCGA-CV-A461 | HNSC | 13.27147877 |
| TCGA-F7-A50J | HNSC | 5.680849296 |
| TCGA-UF-A7JH | HNSC | 14.41778889 |
| TCGA-HD-7753 | HNSC | 5.957557784 |
| TCGA-CN-4730 | HNSC | 10.99633739 |
| TCGA-CV-5439 | HNSC | 10.58975645 |
| TCGA-CQ-6224 | HNSC | 5.734867513 |
| TCGA-BB-4225 | HNSC | 15.54022414 |
| TCGA-CV-A6JY | HNSC | 8.783240471 |
| TCGA-CN-A49B | HNSC | 11.62258268 |
| TCGA-IQ-A61O | HNSC | 1.718114832 |
| TCGA-BA-A4IH | HNSC | 12.77168825 |
| TCGA-CR-7388 | HNSC | 15.78578214 |
| TCGA-MT-A67D | HNSC | 6.159928827 |
| TCGA-CQ-A4C7 | HNSC | 7.216705949 |
| TCGA-CV-7437 | HNSC | 5.29232552 |
| TCGA-IQ-A61H | HNSC | 11.68343643 |
| TCGA-P3-A6T5 | HNSC | 12.69401398 |
| TCGA-QK-AA3K | HNSC | 6.430042132 |
| TCGA-CQ-5323 | HNSC | 18.15176447 |
| TCGA-TN-A7HL | HNSC | 14.94338271 |
| TCGA-CN-A63W | HNSC | 8.916697328 |
| TCGA-CR-7397 | HNSC | 3.600444821 |
| TCGA-CN-A6V7 | HNSC | 8.037840475 |
| TCGA-HD-8634 | HNSC | 6.594706785 |
| TCGA-CN-4738 | HNSC | 8.588471375 |
| TCGA-CN-6013 | HNSC | 5.669188509 |
| TCGA-BB-8596 | HNSC | 6.071964849 |
| TCGA-D6-6824 | HNSC | 4.237415716 |
| TCGA-BA-5151 | HNSC | 4.962178215 |
| TCGA-CR-6492 | HNSC | 6.413413104 |
| TCGA-CV-7091 | HNSC | 7.515480295 |
| TCGA-CV-7261 | HNSC | 18.176078 |
| TCGA-BA-A4II | HNSC | 17.42457665 |
| TCGA-UF-A7JC | HNSC | 9.762310362 |
| TCGA-KU-A6H7 | HNSC | 16.26624682 |
| TCGA-BA-5152 | HNSC | 6.540605516 |
| TCGA-MT-A51W | HNSC | 7.432913278 |
| TCGA-CV-5973 | HNSC | 3.600484103 |
| TCGA-QK-A6VB | HNSC | 16.26773657 |
| TCGA-UF-A71A | HNSC | 13.9875177 |
| TCGA-CV-5976 | HNSC | 13.64641529 |
| TCGA-CV-5970 | HNSC | 4.611622375 |
| TCGA-BB-A5HY | HNSC | 13.93648408 |
| TCGA-BA-A6D8 | HNSC | 4.271017825 |
| TCGA-CN-A642 | HNSC | 10.26292171 |
| TCGA-CV-A465 | HNSC | 4.990473706 |
| TCGA-CV-7440 | HNSC | 4.359096824 |
| TCGA-MT-A67A | HNSC | 3.869875582 |
| TCGA-CR-7385 | HNSC | 21.35144522 |
| TCGA-CR-7394 | HNSC | 4.788527498 |
| TCGA-P3-A5QA | HNSC | 2.904453088 |
| TCGA-CR-5247 | HNSC | 16.43127897 |
| TCGA-CN-4736 | HNSC | 10.44091392 |
| TCGA-BB-4217 | HNSC | 9.985957451 |
| TCGA-CV-7433 | HNSC | 5.424012236 |
| TCGA-T3-A92M | HNSC | 18.47398104 |
| TCGA-H7-A76A | HNSC | 11.97009498 |
| TCGA-HD-A634 | HNSC | 7.306467565 |
| TCGA-CN-A49A | HNSC | 16.55985113 |
| TCGA-IQ-7632 | HNSC | 7.749876277 |
| TCGA-D6-6827 | HNSC | 15.34553874 |
| TCGA-P3-A5QF | HNSC | 20.1192196 |
| TCGA-BA-A6DI | HNSC | 11.62498291 |
| TCGA-CV-7424 | HNSC | 10.81685883 |
| TCGA-CV-7235 | HNSC | 17.33568557 |
| TCGA-CV-A6K2 | HNSC | 9.157497429 |
| TCGA-F7-A61V | HNSC | 15.05377629 |
| TCGA-CV-6938 | HNSC | 5.835745008 |
| TCGA-CQ-6219 | HNSC | 10.67585486 |
| TCGA-CV-5434 | HNSC | 11.35107047 |
| TCGA-CV-A45Y | HNSC | 8.125154681 |
| TCGA-CV-5966 | HNSC | 8.562234337 |
| TCGA-D6-A6ES | HNSC | 4.938123524 |
| TCGA-CR-7376 | HNSC | 3.553465372 |
| TCGA-BA-6872 | HNSC | 9.681223236 |
| TCGA-IQ-A6SH | HNSC | 11.18679166 |
| TCGA-CR-6470 | HNSC | 23.75791293 |
| TCGA-CV-A460 | HNSC | 13.63746759 |
| TCGA-UF-A7JF | HNSC | 8.082537534 |
| TCGA-CN-6023 | HNSC | 10.25911111 |
| TCGA-CR-6477 | HNSC | 8.296381396 |
| TCGA-QK-A6II | HNSC | 8.626863376 |
| TCGA-CR-7389 | HNSC | 6.221063995 |
| TCGA-CV-7180 | HNSC | 7.05651696 |
| TCGA-CV-7406 | HNSC | 10.47735609 |
| TCGA-BA-5555 | HNSC | 15.8990311 |
| TCGA-F7-A61W | HNSC | 13.36695767 |
| TCGA-CX-7219 | HNSC | 6.879146982 |
| TCGA-UP-A6WW | HNSC | 11.60567107 |
| TCGA-QK-A8Z8 | HNSC | 15.55405866 |
| TCGA-T2-A6X2 | HNSC | 15.51126766 |
| TCGA-4P-AA8J | HNSC | 7.683546653 |
| TCGA-BA-5557 | HNSC | 5.648322766 |
| TCGA-CR-7402 | HNSC | 25.68439098 |
| TCGA-CQ-6228 | HNSC | 19.29000988 |
| TCGA-CN-4737 | HNSC | 8.101632054 |
| TCGA-UF-A7JD | HNSC | 7.264428757 |
| TCGA-P3-A5Q6 | HNSC | 8.866229008 |
| TCGA-CV-7425 | HNSC | 10.9286933 |
| TCGA-CN-6022 | HNSC | 12.00918251 |
| TCGA-CR-6480 | HNSC | 20.2285424 |
| TCGA-F7-8298 | HNSC | 18.03879853 |
| TCGA-BA-4074 | HNSC | 4.891395078 |
| TCGA-CN-6988 | HNSC | 10.75050099 |
| TCGA-CV-A6JD | HNSC | 6.516740915 |
| TCGA-F7-A50I | HNSC | 46.52706939 |
| TCGA-DQ-5630 | HNSC | 16.29207872 |
| TCGA-D6-6823 | HNSC | 11.70147396 |
| TCGA-CQ-5333 | HNSC | 5.157103366 |
| TCGA-CV-7103 | HNSC | 4.073135899 |
| TCGA-CV-A45R | HNSC | 9.420750298 |
| TCGA-CQ-5331 | HNSC | 7.268446779 |
| TCGA-D6-A6EM | HNSC | 7.299148478 |
| TCGA-CR-7372 | HNSC | 8.666542539 |
| TCGA-T2-A6X0 | HNSC | 40.45008086 |
| TCGA-HL-7533 | HNSC | 12.37769771 |
| TCGA-H7-8502 | HNSC | 16.19395471 |
| TCGA-BA-A8YP | HNSC | 18.3786931 |
| TCGA-KU-A6H8 | HNSC | 4.533984629 |
| TCGA-CV-6950 | HNSC | 7.264038169 |
| TCGA-CN-5370 | HNSC | 9.150913449 |
| TCGA-CV-6933 | HNSC | 22.63781082 |
| TCGA-UF-A718 | HNSC | 12.70398147 |
| TCGA-CR-5243 | HNSC | 20.6293777 |
| TCGA-BB-4224 | HNSC | 30.55719287 |
| TCGA-CV-A6JU | HNSC | 15.17697592 |
| TCGA-CV-6937 | HNSC | 16.06385251 |
| TCGA-H7-8501 | HNSC | 10.12446793 |
| TCGA-CV-7435 | HNSC | 5.582644895 |
| TCGA-HD-A6HZ | HNSC | 8.000254575 |
| TCGA-CV-A6JE | HNSC | 7.17600758 |
| TCGA-CN-A6V6 | HNSC | 12.70598858 |
| TCGA-CR-6474 | HNSC | 4.922082491 |
| TCGA-BA-5558 | HNSC | 5.407981318 |
| TCGA-CR-7373 | HNSC | 6.348467335 |
| TCGA-CR-7398 | HNSC | 14.41871185 |
| TCGA-CV-7247 | HNSC | 9.91217243 |
| TCGA-CV-7422 | HNSC | 11.55868843 |
| TCGA-RS-A6TP | HNSC | 16.51271389 |
| TCGA-CN-5369 | HNSC | 10.63728946 |
| TCGA-CV-7250 | HNSC | 7.573892465 |
| TCGA-CN-4731 | HNSC | 15.90164959 |
| TCGA-DQ-5625 | HNSC | 9.392474165 |
| TCGA-H7-7774 | HNSC | 8.514350217 |
| TCGA-D6-6516 | HNSC | 5.705658601 |
| TCGA-QK-A64Z | HNSC | 7.732096678 |
| TCGA-BA-7269 | HNSC | 6.490480172 |
| TCGA-CV-5440 | HNSC | 28.38454648 |
| TCGA-CV-A45W | HNSC | 15.89672879 |
| TCGA-CN-6989 | HNSC | 7.222656356 |
| TCGA-CN-5359 | HNSC | 7.374552683 |
| TCGA-CR-6481 | HNSC | 11.73600724 |
| TCGA-CV-7446 | HNSC | 11.02587194 |
| TCGA-CN-5365 | HNSC | 13.24031961 |
| TCGA-F7-A624 | HNSC | 5.157503704 |
| TCGA-CQ-6220 | HNSC | 12.94233965 |
| TCGA-CN-5363 | HNSC | 14.50623689 |
| TCGA-T2-A6WX | HNSC | 7.885549878 |
| TCGA-CQ-7068 | HNSC | 12.7376273 |
| TCGA-CV-A6K1 | HNSC | 9.194249417 |
| TCGA-HD-7832 | HNSC | 14.59974739 |
| TCGA-MZ-A6I9 | HNSC | 12.57409685 |
| TCGA-CV-7416 | HNSC | 6.451919483 |
| TCGA-D6-8568 | HNSC | 9.253574767 |
| TCGA-HD-8224 | HNSC | 14.07072649 |
| TCGA-CQ-A4CG | HNSC | 9.618206907 |
| TCGA-CN-A49C | HNSC | 23.55028342 |
| TCGA-D6-A6EQ | HNSC | 5.733972761 |
| TCGA-CQ-A4C9 | HNSC | 16.94022043 |
| TCGA-CR-7404 | HNSC | 18.74142961 |
| TCGA-CR-7382 | HNSC | 4.347514618 |
| TCGA-CV-7427 | HNSC | 5.636112404 |
