# Supplementary material for: Comprehensive analysis of macrophage-related multigene signature in the tumor microenvironment of head and neck squamous cancer
Source: Aging (Albany NY). 2021 Feb 11;13(4):5718–47. doi: 10.18632/aging.202499 (PMC7950226; doi:10.18632/aging.202499)
Supplement: Supplementary Tables 4 and 5 [file aging-13-202499-s004.pdf]

SUPPLEMENTARY TABLES

Supplementary Table 4. Expression of FANCE in paired samples.

Patients	Types	Expression of FANCE
TCGA-CV-6933	Normal	1.529861739
TCGA-CV-6933	HNSC	22.63781082
TCGA-CV-6934	Normal	6.58527099
TCGA-CV-6934	HNSC	5.345447825
TCGA-CV-6935	Normal	5.652059057
TCGA-CV-6935	HNSC	16.61917616
TCGA-CV-6936	Normal	4.78916536
TCGA-CV-6936	HNSC	9.993552436
TCGA-CV-6938	Normal	5.641356245
TCGA-CV-6938	HNSC	5.835745008
TCGA-CV-6939	Normal	1.339942754
TCGA-CV-6939	HNSC	19.08754488
TCGA-CV-6943	Normal	4.338543649
TCGA-CV-6943	HNSC	5.036290608
TCGA-CV-6955	Normal	5.386422691
TCGA-CV-6955	HNSC	5.692342856
TCGA-CV-6956	Normal	5.715106667
TCGA-CV-6956	HNSC	20.69060622
TCGA-CV-6959	Normal	5.670946031
TCGA-CV-6959	HNSC	12.70210582
TCGA-CV-6960	Normal	7.561391089
TCGA-CV-6960	HNSC	7.025494634
TCGA-CV-6961	Normal	4.013741188
TCGA-CV-6961	HNSC	8.670047656
TCGA-CV-6962	Normal	7.272354399
TCGA-CV-6962	HNSC	14.70198091
TCGA-CV-7091	Normal	1.386408086
TCGA-CV-7091	HNSC	7.515480295
TCGA-CV-7097	Normal	2.551280952
TCGA-CV-7097	HNSC	10.65048969
TCGA-CV-7101	Normal	8.43816359
TCGA-CV-7101	HNSC	11.00130434
TCGA-CV-7103	Normal	5.686144365
TCGA-CV-7103	HNSC	4.073135899
TCGA-CV-7177	Normal	6.448477905
TCGA-CV-7177	HNSC	9.111828696
TCGA-CV-7178	Normal	5.380158915
TCGA-CV-7178	HNSC	11.71185774
TCGA-CV-7183	Normal	6.36133641
TCGA-CV-7183	HNSC	6.631037981
TCGA-CV-7235	Normal	2.977478339
TCGA-CV-7235	HNSC	17.33568557
TCGA-CV-7238	Normal	2.669154294
TCGA-CV-7238	HNSC	4.389969145
TCGA-CV-7242	Normal	4.359282565
TCGA-CV-7242	HNSC	9.362439224
TCGA-CV-7245	Normal	2.215915124
TCGA-CV-7245	HNSC	7.177659298
TCGA-CV-7250	Normal	4.41484975

TCGA-CV-7250	HNSC	7.573892465
TCGA-CV-7252	Normal	4.581651943
TCGA-CV-7252	HNSC	10.66893562
TCGA-CV-7255	Normal	6.993466272
TCGA-CV-7255	HNSC	11.324035
TCGA-CV-7261	Normal	11.11876024
TCGA-CV-7261	HNSC	18.176078
TCGA-CV-7406	Normal	2.328782586
TCGA-CV-7406	HNSC	10.47735609
TCGA-CV-7416	Normal	4.504562016
TCGA-CV-7416	HNSC	6.451919483
TCGA-CV-7423	Normal	4.886702718
TCGA-CV-7423	HNSC	5.58256426
TCGA-CV-7424	Normal	6.478547809
TCGA-CV-7424	HNSC	10.81685883
TCGA-CV-7425	Normal	6.08632076
TCGA-CV-7425	HNSC	10.9286933
TCGA-CV-7432	Normal	3.016398651
TCGA-CV-7432	HNSC	11.44222552
TCGA-CV-7434	Normal	2.242350326
TCGA-CV-7434	HNSC	7.879035693
TCGA-CV-7437	Normal	6.584120952
TCGA-CV-7437	HNSC	5.29232552
TCGA-CV-7438	Normal	6.272020205
TCGA-CV-7438	HNSC	8.1586412
TCGA-CV-7440	Normal	5.641184068
TCGA-CV-7440	HNSC	4.359096824
TCGA-H7-A6C4	Normal	2.469733377
TCGA-H7-A6C4	HNSC	6.266125779
TCGA-H7-A6C5	Normal	3.625142974
TCGA-H7-A76A	HNSC	11.97009498
TCGA-HD-8635	Normal	4.885357822
TCGA-HD-8635	HNSC	7.819542853
TCGA-HD-A6HZ	Normal	3.182422999
TCGA-HD-A6HZ	HNSC	8.000254575
TCGA-HD-A6I0	Normal	1.962784244
TCGA-HD-A6I0	HNSC	7.497811298
TCGA-KU-A6H7	Normal	30.9665178
TCGA-KU-A6H7	HNSC	16.26624682
TCGA-UF-A71A	Normal	17.20650691
TCGA-UF-A71A	HNSC	13.9875177
TCGA-WA-A7GZ	Normal	4.531920239
TCGA-WA-A7GZ	HNSC	16.66120059

Supplementary Table 5. Relationship between methylation sites and expression of FANCE.

Methylation sites	Cor.	*P*
cg18744234	-0.469	$6.45×10^{-30}$
cg03030757	-0.356	$5.61×10^{-17}$
cg09490277	0.192	$9.71×10^{-6}$
cg15267307	-0.155	$3.84×10^{-4}$
cg12798052	-0.151	$5.26×10^{-4}$
cg05261496	-0.084	0.056
cg17803089	0.053	0.228
cg19335943	0.04	0.366
cg08672023	-0.037	0.394
cg27198948	0.006	0.889
